# An altered oral microbiota induced by injections of superparamagnetic iron oxide nanoparticle‐labeled periodontal ligament stem cells helps periodontal bone regeneration in rats

**DOI:** 10.1002/btm2.10466

**Published:** 2022-12-13

**Authors:** Zihan Shi, Lu Jia, Qian Zhang, Liuxu Sun, Xinyue Wang, Xuan Qin, Yang Xia

**Affiliations:** ^1^ Jiangsu Key Laboratory of Oral Diseases Nanjing Medical University Nanjing Jiangsu People's Republic of China; ^2^ Jiangsu Province Engineering Research Center of Stomatological Translational Medicine Nanjing Medical University Nanjing Jiangsu People's Republic of China; ^3^ Department of Emergency General Dentistry, Hebei Key Laboratory of Stomatology Hebei Medical University Shijiazhuang Hebei People's Republic of China; ^4^ Suzhou Stomatological Hospital Suzhou Jiangsu People's Republic of China

**Keywords:** *Lactobacillus reuteri*, oral microbiota, periodontal ligament stem cells, periodontal regeneration, probiotics, superparamagnetic iron oxide nanoparticles

## Abstract

Stem cell injection is good for periodontal regeneration due to the capacity of stem cells to differentiate toward osteogenic direction and to regulate the production of pro‐ and anti‐inflammatory cytokines. However, injected cells are difficult to track in vivo. And there is microbiota in oral cavity, the dysbiosis of which leads to the damage and loss of periodontal tissue. Here, we demonstrated an enhanced periodontal repair was due to an altered oral microbiota. Periodontal defects were surgically prepared in rats, and periodontal ligament stem cells (PDLSCs) labeled by superparamagnetic iron oxide (SPIO) nanoparticles (PC‐SPIO) were injected, with PDLSCs and saline treatments as controls. Detected by magnetic resonance imaging (MRI) and histological staining, PC‐SPIO was major at limited areas in regenerated periodontal tissues. PC‐SPIO‐treated rats achieved better periodontal regeneration than the other two groups. Concurrently, the oral microbiota of PC‐SPIO‐treated rats was changed, presenting SPIO‐Lac as a biomarker. SPIO‐Lac assisted periodontal repair in vivo, inhibited the inflammation of macrophages induced by lipopolysaccharide (LPS) and antibacterial in vitro. Therefore, our study proved that SPIO‐labeled cells can be tracked in periodontal defect and highlighted a potential positive role of an oral microbiota in periodontal regeneration, suggesting the possibility of periodontal repair promotion by manipulating oral microbiota.

## 1 INTRODUCTION

Regeneration of intraosseous defects is an important therapeutic goal of periodontal treatment, because the existence of deep intraosseous defects creates a high risk of recrudescence and further progression of periodontitis. Bone graft replacement is a classical treatment that has been used commonly in the clinic.^1^, ^2^ However, it is associated with problems such as morbidity caused by a second surgical site and the possibility of disease transmission. Moreover, the patients suffer trauma, pain, and swelling because of the collection of autogenous bone and the implantation of bone grafts.^3^ Stem cell therapy brings hope to patients suffering from periodontal defects.

Mesenchymal stem cells (MSCs), which have been used to promote periodontal regeneration, include bone marrow‐derived MSCs (BMMSCs), adipose tissue‐derived MSCs, periodontal ligament stem cells (PDLSCs), dental pulp‐derived MSCs (DPMSCs), dental epithelial stem cells, stem cells from the dental apical papilla (SCAPs), and stem cells from the dental follicle (DFSCs).^4^, ^5^, ^6^, ^7^, ^8^, ^9^ The application of all these MSCs has achieved positive results in periodontal regeneration.^4^, ^5^, ^6^, ^7^, ^8^, ^9^ However, PDLSCs are considered the best choice among them.

PDLSCs possess low immunogenicity and can be obtained noninvasively from periodontal tissue during standard dental scaling and root planning (SRP) procedures. They present a similar phenotypic profile to BMMSCs, indicating a strong osteogenic potential. In addition, they are fast growing, providing the possibility of ex vivo expansion.^10^ Moreover, signals from the transplanted PDLSCs might alter the immune microenvironment to enhance periodontal regeneration.^11^ When transplanted into periodontal defects, PDLSCs have the potential to form bone, cementum, and periodontal ligament‐like structures, while BMMSCs mainly produce bone and bone‐marrow‐like structures.^12^

Compared with other stem cell delivery strategies used to treat periodontal bone defects, such as cell sheets and cell‐seeded scaffolds, cell injection has its own advantages. Although not all the injected cells can reach the injured site, injection can avoid the trauma caused by surgery.^8^ Moreover, this minimally invasive technique can be repeated until periodontal healing reaches the required condition.^13^

Stem cell injection is good for periodontal regeneration.^4^, ^6^, ^8^ Underlying mechanisms have been explored from their capacities to differentiate toward the osteogenic direction and to regulate the production of pro‐ and anti‐inflammatory cytokines.^4^, ^6^, ^8^ However, periodontal defects heal in the oral cavity, which is colonized by billions of bacteria, fungi, and viruses, known as the oral microbiota. Homeostasis of the oral microbiota plays a crucial role in maintaining the well‐being and healthy status of the human host.^14^ Perturbations in the microbiota caused by certain factors, such as dental plaque accumulation, which is a sticky film composed mainly of bacteria, can lead to the initiation and development of periodontitis. Correspondingly, dysbiosis, which is the shift of species and functions associated with diseases, is also observed in periodontitis.^15^ These changes to the microbiota mainly comprise increases in pathogenic bacteria, which amplify the inflammatory response by activating the host immune response, beginning a cycle of microbiota changes and enhanced inflammation, leading eventually to loss of tissue integrity, attachment, and bone loss.^16^, ^17^

Although has been notorious for the damaging effect, it is still possible that a distinct oral microbiota plays a positive role for periodontal regeneration. We previously found that superparamagnetic iron oxide (SPIO)‐coated scaffolds induced better palate‐bone regeneration than the uncoated controls, and the effect was partly related to alteration of the oral microbiota caused by the antibacterial effects of SPIO.^18^ And topical treatment with probiotics has protective effect against periodontitis.^19^ These results suggested that certain alteration of oral microbiota may be beneficial to bone regeneration.

For cell function enhancements and cell labeling, SPIO nanoparticles have great potential to modify PDLSCs. SPIO nanoparticles containing an iron oxide core (7 nm in diameter) and a polyglucose sorbitol carboxymethyether (PSC) shell (20 nm in thickness) comprise the FDA‐approved nanodrug ferumxytol.^20^ They are biosafe and have been applied clinically as an iron supplement and a magnetic resonance imaging (MRI) T2 contrast agent.^21^ Our group developed an SPIO nanoparticle, whose structure mimics ferumxytol.^20^ Previously, we used gelatin sponge scaffolds labeled with this SPIO nanoparticle to fill mandible defects after the extraction of rat incisors and found a significant increase in bone regeneration and a decrease in signal intensity of T2‐weighted MRI in the SPIO‐labeled scaffold group.^22^ During the healing period, changes in the image intensity of the scaffolds, which indicated scaffold degradation and interaction with host tissues, could be monitored visually.^22^ Recently, this SPIO nanoparticle was applied for neural stem cell labeling under ultrasonic exposure.^23^ Meanwhile, SPIO‐labeled cells stain positively with Prussian blue, facilitating supplementary histological assessments. In addition, this SPIO nanoparticle can enhance the osteogenic differentiation of human BMMSCs and human DPMSCs.^24^, ^25^ Moreover, it can drive macrophage transformation toward an M1‐like phenotype to inhibit tumor growth,^26^ and increase the level of interleukin‐10 (IL‐10) in liver macrophages, leading to inhibition of inflammation in lipopolysaccharide (LPS)‐induced sepsis and liver injury.^27^

In this study, SPIO‐labeled PDLSCs (PC‐SPIO) were repeatedly injected into inflammatory periodontal defect in rats, using injections of PDLSCs and saline as controls. The injected PC‐SPIO was tracked by MRI in vivo and histological staining, and the periodontal regeneration by PC‐SPIO was detected. Moreover, the oral microbiota of rats injected with PC‐SPIO was changed, and the local application of the biomarker from changed oral microbiota could enhance periodontal regeneration. This study represents the first exploration of the positive role of an altered oral microbiota in periodontal regeneration.

## 2 RESULTS

### 2.1 SPIO labeling of PDLSCs did not damage cell viability and made them MRI trackable

**FIGURE 1:**
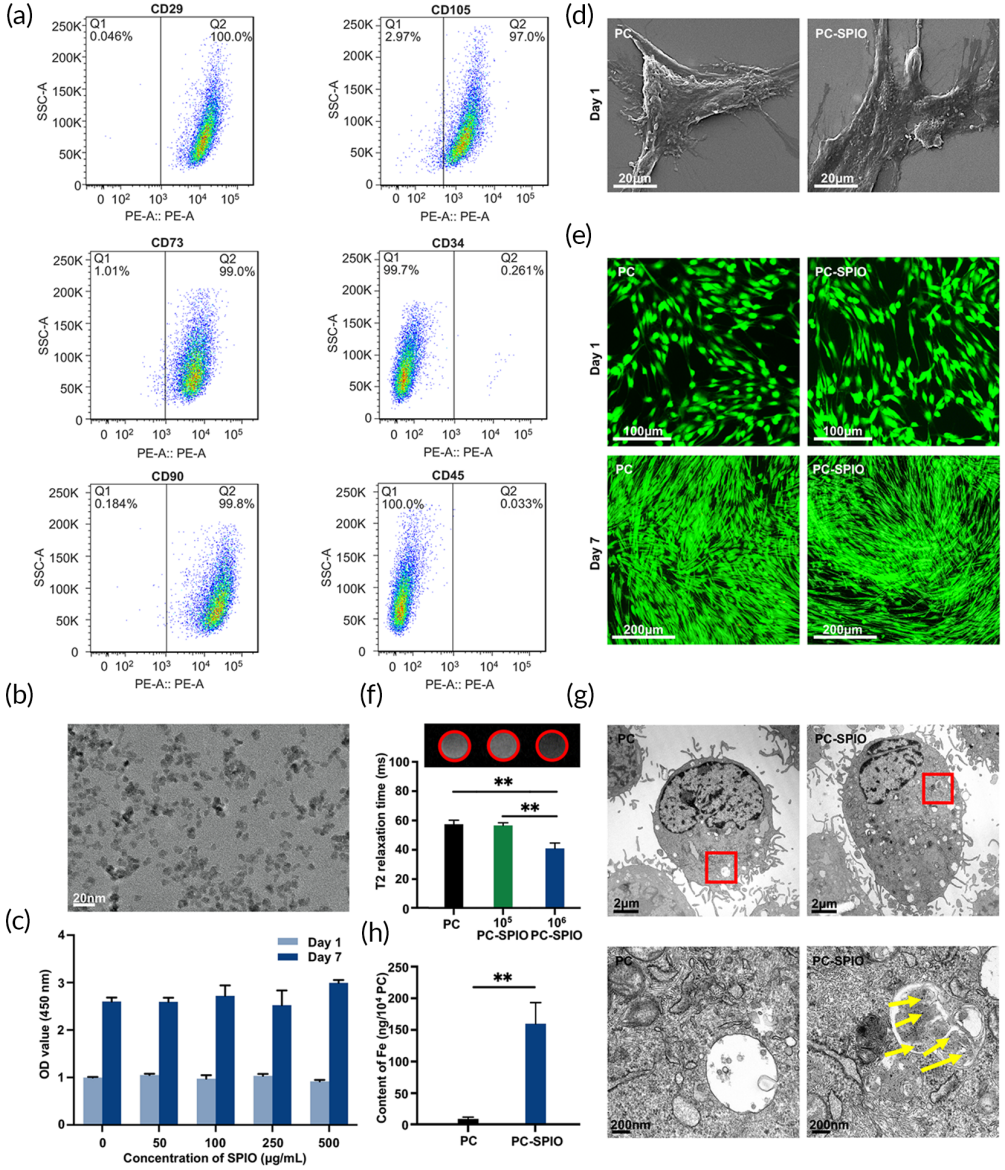
(a) Flow cytometry analysis of the cells isolated from human periodontal ligaments. (b) Transmission electron microscope (TEM) image of superparamagnetic iron oxide (SPIO) nanoparticles. (c) Cell viability of periodontal ligament stem cells (PDLSCs) treated with different concentrations of SPIO nanoparticles. (d) SEM images of PC and SPIO‐labeled PDLSCs (PC‐SPIO) at 1 day (×3000). (e) Live/dead staining of PC and PC‐SPIO at 1 day (×200) and 7 days (×100). (f) In vitro MRI images of different concentrations of PC and PC‐SPIO and their T2 relaxation time. (g) TEM images of PC and PC‐SPIO. SPIO nanoparticles are indicated by yellow arrows. (h) Measurement of the iron content in PC and PC‐SPIO using ICP‐OES. PC represents PDLSCs, and PC‐SPIO represents PDLSCs treated with 250 μg/ml SPIO nanoparticles for 24 h. (*n* = 3) (*p < 0.05, **p < 0.01)

The cells isolated from human periodontal ligaments strongly expressed markers CD29 (99.8%), CD73 (99.6%), CD90 (99.8%), and CD105 (98.7%) and exhibited low expressions of CD34 (0.21%), and CD45 (0.22%). Therefore, they were identified as PDLSCs (Figure 1a). SPIO nanoparticles were uniformly dispersed in aqueous solution with mean diameter of 7–10 nm (Figure 1b). The viability of PDLSCs was not affected when concentrations of SPIO nanoparticles up to 500 μg/ml (*p* > 0.05; Figure 1c). Therefore, PDLSCs were cultured with 250 μg/ml SPIO nanoparticles and termed as PC‐SPIO, using PDLSCs (termed as PC) as a control. Cell morphology under SEM (×3000) proved that both PC and PC‐SPIO spread well at 1 day (Figure 1d). At low magnification, they were homogenous bipolar spindle‐like cells, presenting a fibroblastic‐like shape at 1 day (×200). And they proliferated well, showing dense green fluorescent staining at 7 days (×100) (Figure 1e).

In in vitro labeling test, no significant difference was detected between PC and 1 × 10^5^/ml PC‐SPIO, both of which appeared as bright white circles (Figure 1f). Comparatively, the image of 1 × 10^6^/ml PC‐SPIO was significantly different from the above two and produced good contrast, presenting as a dark black circle (Figure 1f). T2‐mapping results were consistent with the images, showing that the T2 relaxation time of 1 × 10^6^/ml PC‐SPIO was significantly shorter than that of PC and 1 × 10^5^/ml PC‐SPIO (*p* < 0.01; Figure 1f). SPIO nanoparticles (marked by yellow arrows in Figure 1g) were detected in PC‐SPIO by transmission electron microscope (TEM). The results of inductively coupled plasma optical emission spectrometer (ICP‐OES) showed that the iron content of PC‐SPIO was significantly higher than that of PC (*p* < 0.01; Figure 1h). Therefore, 1 × 10^6^/ml PC‐SPIO was used for the in vivo injection, using PC and Saline injection as controls.

### 2.2 SPIO nanoparticles labeled PDLSCs can be tracked in vivo

**FIGURE 2:**
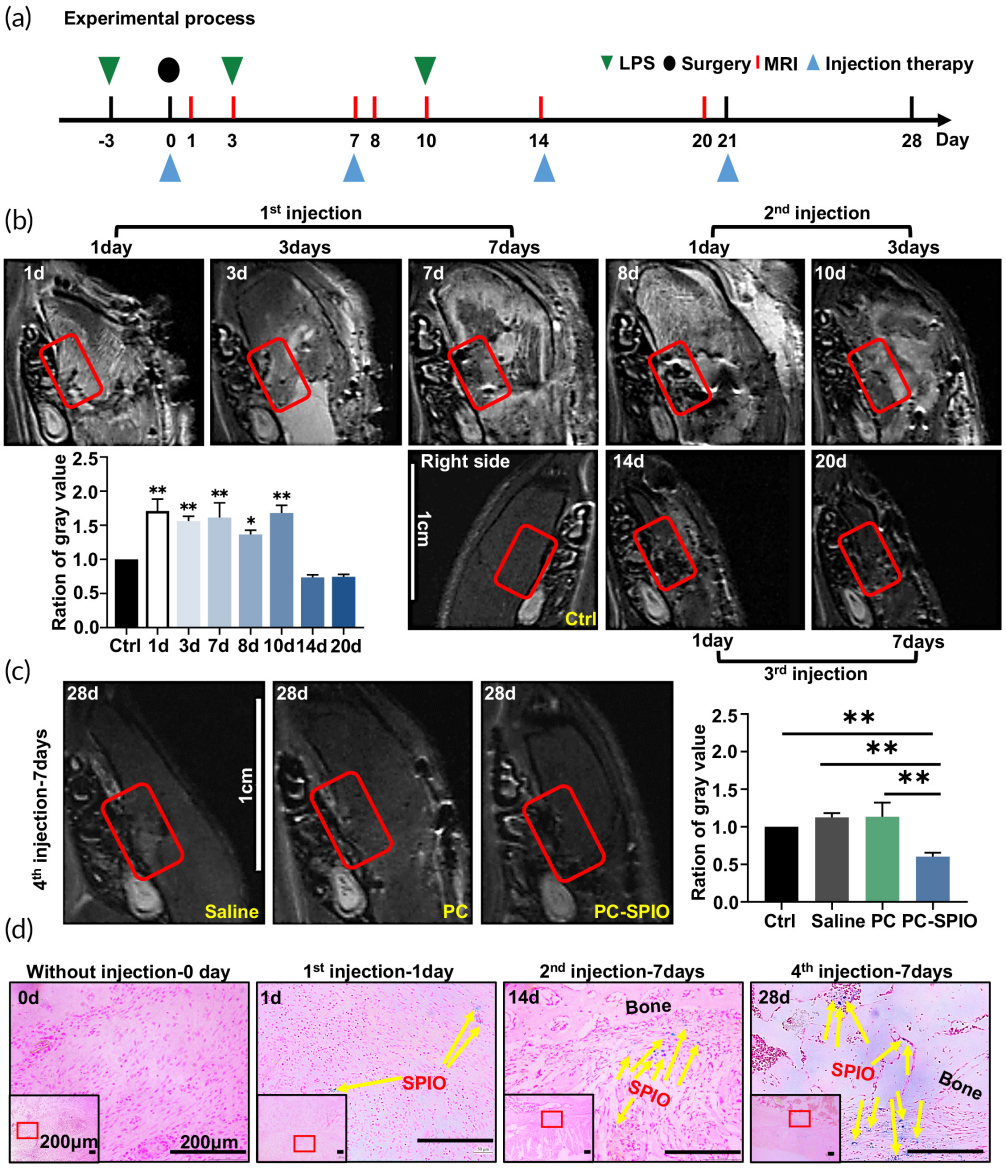
(a) Time sequence of animal experiments, including lipopolysaccharide (LPS) injections, surgery, magnetic resonance imaging (MRI) detection, and injection therapy. (b) In vivo MRI detection of periodontal defects after one, two, and three SPIO‐labeled PDLSCs (PC‐SPIO) injections. (c) In vivo MRI detection of periodontal defects at 7 days after four injections with Saline, PC, and PC‐SPIO. (d) Prussian blue staining of tissues from the periodontal defect area. (*n* = 3) (*p < 0.05, **p < 0.01)

The process of the animal experiments included one surgery, three LPS injections, and four injection treatments (Figure 2a). MRI was performed on rats to track the PC‐SPIO in vivo (Figure 2b,c). The red boxes, which were used for analysis, represent the area of the periodontal defect including some adjacent buccal soft tissue. The similar position on the contralateral side was used as a control. In PC‐SPIO group, the thickness from the molar to the epidermis increased from 1 to 8 days because of the inflammation and swelling from the surgery. It gradually became thinner from 10 days and almost returned to normal thickness at 14 days. The defect area was brighter than the control from 1 to 10 days and presented normal brightness after 10 days. The image was much brighter than the control after surgery and the first injection. Then, the images turned darker after the second injection but became brighter with time. However, the brightness did not change much after the third injection, possibly because of the obvious new bone regeneration. When setting the gray value ratio of the control as 1 for the quantitative assessment, it was found that the ratio was significantly greater than 1 from 1 to 10 days (*p <* 0.01) and was less than 1 from 14 days (*p >* 0.05; Figure 2b).

After the fourth injection, the image of PC‐SPIO group was darker than that of the Saline group, PC group, and the control side at 28 days owing to existence of sufficient PC‐SPIO nanoparticles and satisfactory regeneration of the defect (Figure 2c). Consistently, the gray value ratio of PC‐SPIO group was significantly lower than that of the other two groups and the control (*p <* 0.01; Figure 2c).

Images of Prussian blue staining showed that no positive area was detected in tissues after surgery without PC‐SPIO injection. A few blue spots, indicating SPIO nanoparticles, were observed in the buccal soft tissue of the defect at 1 day after the first injection of PC‐SPIO. More blue spots that were closer to the bone defect were observed with repeated injection of PC‐SPIO over time, and the increase was significant (*p <* 0.01; Figures 2d and S1). The blue staining extended deep into the bone defect area and throughout the buccal soft tissue (Figure 2d), which might be related to the regulation of the oral microbiota and the formation of new bone.

### 2.3 PC‐SPIO injection promoted periodontal bone regeneration

**FIGURE 3:**
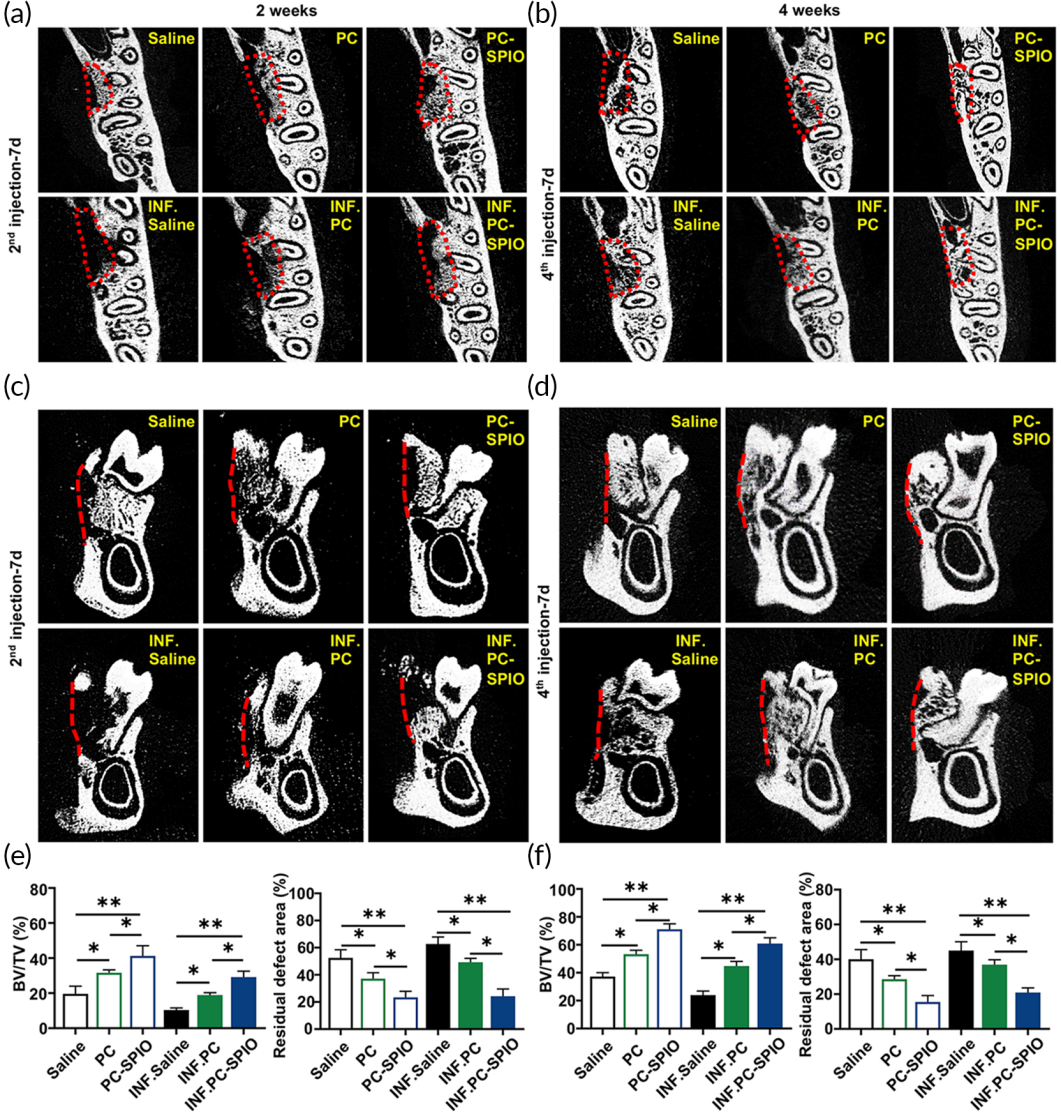
(a) Evaluation of periodontal bone regeneration using micro‐CT at 2 weeks after surgery (transverse planes). (b) Evaluation of periodontal bone regeneration by micro‐CT at 4 weeks after surgery (transverse planes). (c) Evaluation of periodontal bone regeneration using micro‐CT at 2 weeks after surgery (coronary planes). (d) Evaluation of periodontal bone regeneration by micro‐CT at 4 weeks after surgery (coronary planes). (e) Quantitative analysis of periodontal bone regeneration by micro‐CT, including BV/TV and the residual defect area, at 2 weeks after surgery. (f) Quantitative analysis of periodontal bone regeneration by micro‐CT, including BV/TV and the residual defect area, at 4 weeks after surgery. (*n* = 3) (*p < 0.05, **p < 0.01)

**FIGURE 4:**
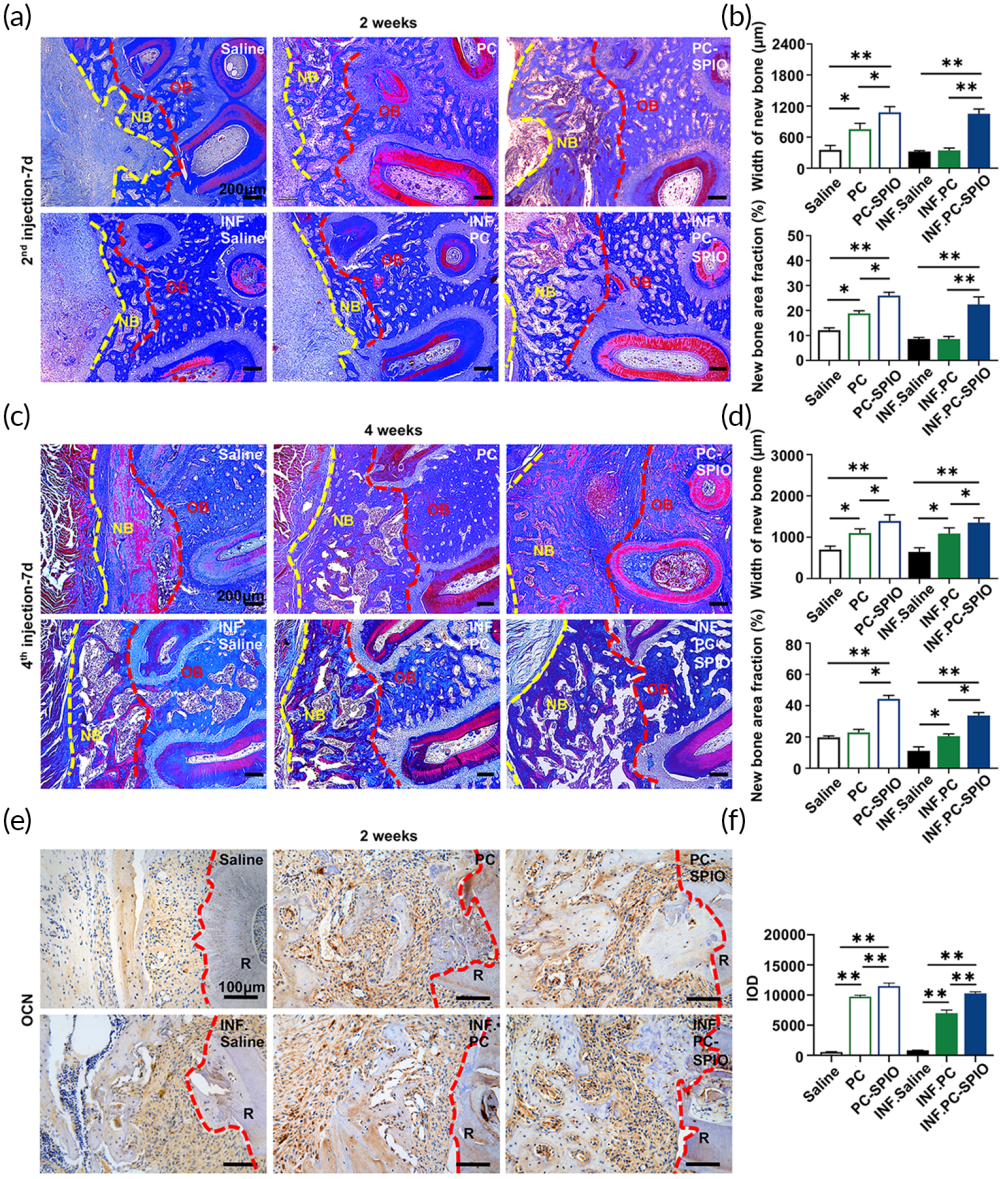
(a) Evaluation of periodontal bone regeneration by Masson staining at 2 weeks after surgery. (b) Quantitative analysis of periodontal bone regeneration by Masson staining, including the width of the buccal new bone and new bone area fraction, at 2 weeks after surgery. (c) Evaluation of periodontal bone regeneration by Masson staining at 4 weeks after surgery. (d) Quantitative analysis of periodontal bone regeneration by Masson staining, including the width of the buccal new bone and new bone area fraction, at 4 weeks after surgery. NB, new bone; OB, original bone. (e) Immunohistochemical staining of osteocalcin (OCN) in the defect area at 2 weeks. (f) Quantitative evaluation of the integral optical density (IOD) of OCN. (*n* = 3) (*p < 0.05, **p < 0.01)

The mandibles were collected and evaluated using micro‐CT (Figure 3) and histological staining (Figure 4) at 2 and 4 weeks after surgery when they had received two or four injections, respectively. Generally, poor, or low levels of regeneration were observed in groups treated with LPS compared with those without, indicating that LPS prevented tissue healing and induced tissue inflammation (Figure S2).

Micro‐CT indicated that the repair of periodontal defect was in the sequence: PC‐SPIO group > PC group > Saline group, with the repair being not only more extensive but also better calcified at 2 and 4 weeks post‐surgery (Figure 3). The trabecular bone volume (BV/TV), a higher value of which represents better periodontal bone regeneration, values were in the sequence: PC‐SPIO group > PC group > Saline group at 2 and 4 weeks post‐surgery (*p* < 0.05). While another index, the residual bone defect area, showed the opposite order (*p* < 0.05). Histological staining, including hematoxylin and eosin (H&E) staining (Figure S3) and Masson staining (Figure 4a–d), was performed to confirm the radiological findings. Consistent with micro‐CT results, the PC‐SPIO group had thicker and denser new bone than the other two groups at 2 and 4 weeks, with and without LPS (*p* < 0.05). In addition, new bone formation in the PC group was better than that in the Saline group (*p* < 0.05; Figure 4a–d). These results indicated that the new bone in the PC‐SPIO group was superior to that in the other two groups, not only in quality but also in quantity.

Immunohistochemical (IHC) staining was performed to detect the expression of osteocalcin (OCN) (Figure 4e,f). OCN expression was significantly increased in PC‐SPIO group compared with Saline and PC groups with and without LPS at 2 weeks (*p* < 0.01). Moreover, OCN expression in PC groups was higher than in Saline groups with and without LPS at 2 weeks (*p* < 0.01). These results proved that PC‐SPIO injection achieved better bone regeneration in rats' periodontal defect and presented stronger osteogenic property, while no obvious adverse changes of rats' major organs were observed in PC‐SPIO group (Figure S4).

### 2.4 Pro‐inflammatory cytokines were decreased and anti‐inflammatory cytokines were increased in response to PC‐SPIO injection in vivo

**FIGURE 5:**
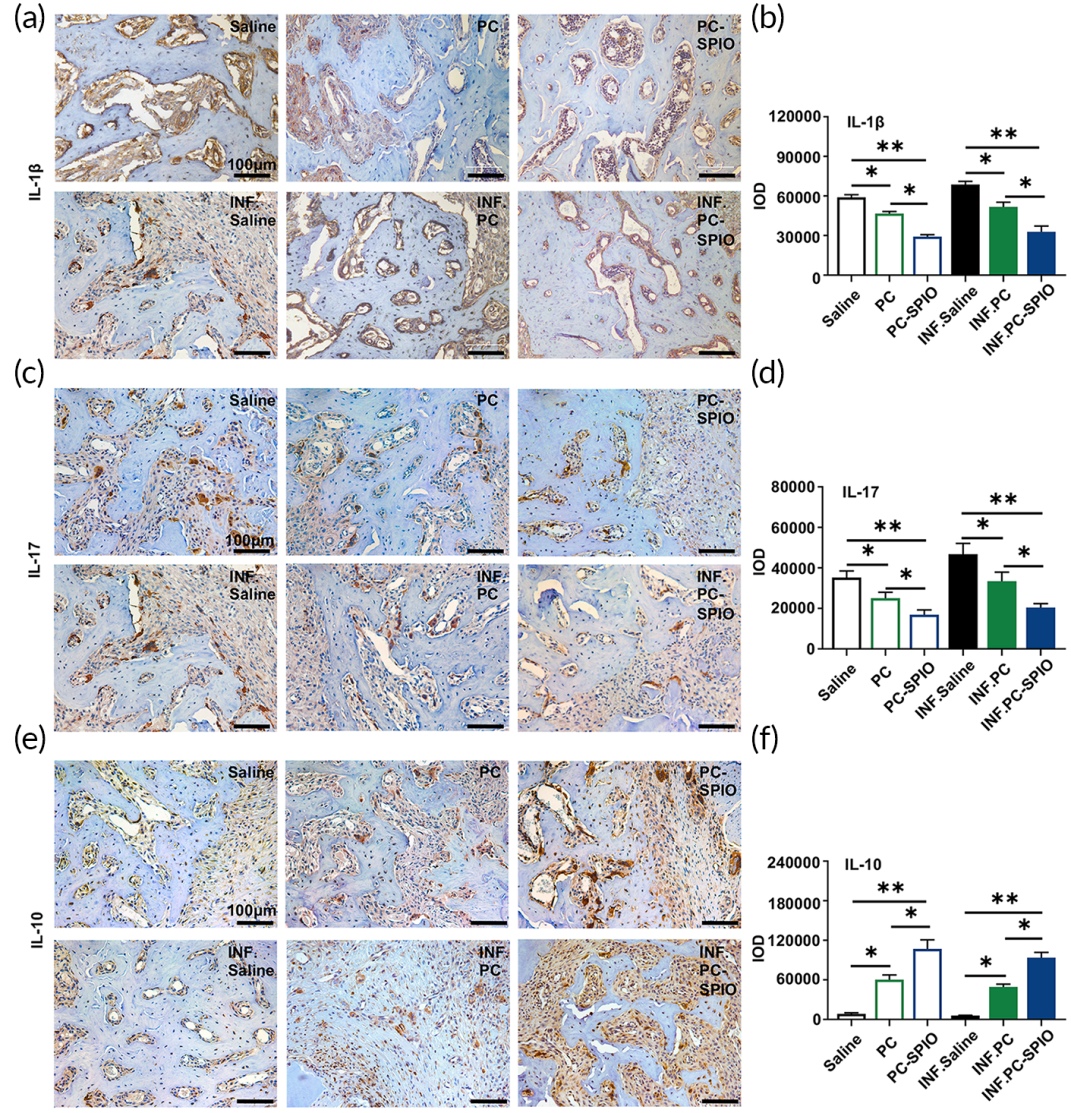
(a) Immunohistochemical staining of IL‐1β in the defect area at 2 weeks. (b) Quantitative evaluation of the integral optical density (IOD) of IL‐1β. (c) Immunohistochemical staining of IL‐17 in the defect area at 2 weeks. (d) Quantitative evaluation of the IOD of IL‐17. (e) Immunohistochemical staining of IL‐10 in the defect area at 2 weeks. (f) Quantitative evaluation of the IOD of IL‐10. (*n* = 3) (*p < 0.05, **p < 0.01)

The groups treated with LPS showed higher contents of IL‐1β and IL‐17, while the PC‐SPIO group had the lowest IL‐1β and IL‐17 contents and the highest IL‐10 content compared with those of the Saline and PC groups (*p* < 0.05; Figure 5). PC group presented lower IL‐1β and IL‐17 levels, and higher IL‐10 levels than Saline group (*p* < 0.05; Figure 5). The levels of IL‐1β and IL‐17 correlated positively with an inflammatory effect, while IL‐10 correlated positively with an anti‐inflammatory effect.^28^, ^29^, ^30^ Therefore, these results indicated that periodic injection of PC‐SPIO significantly inhibited the inflammatory reaction, mainly by decreasing IL‐1β and IL‐17 levels and increasing IL‐10 levels.

### 2.5 The oral microbiota changed in response to different treatments

**FIGURE 6:**
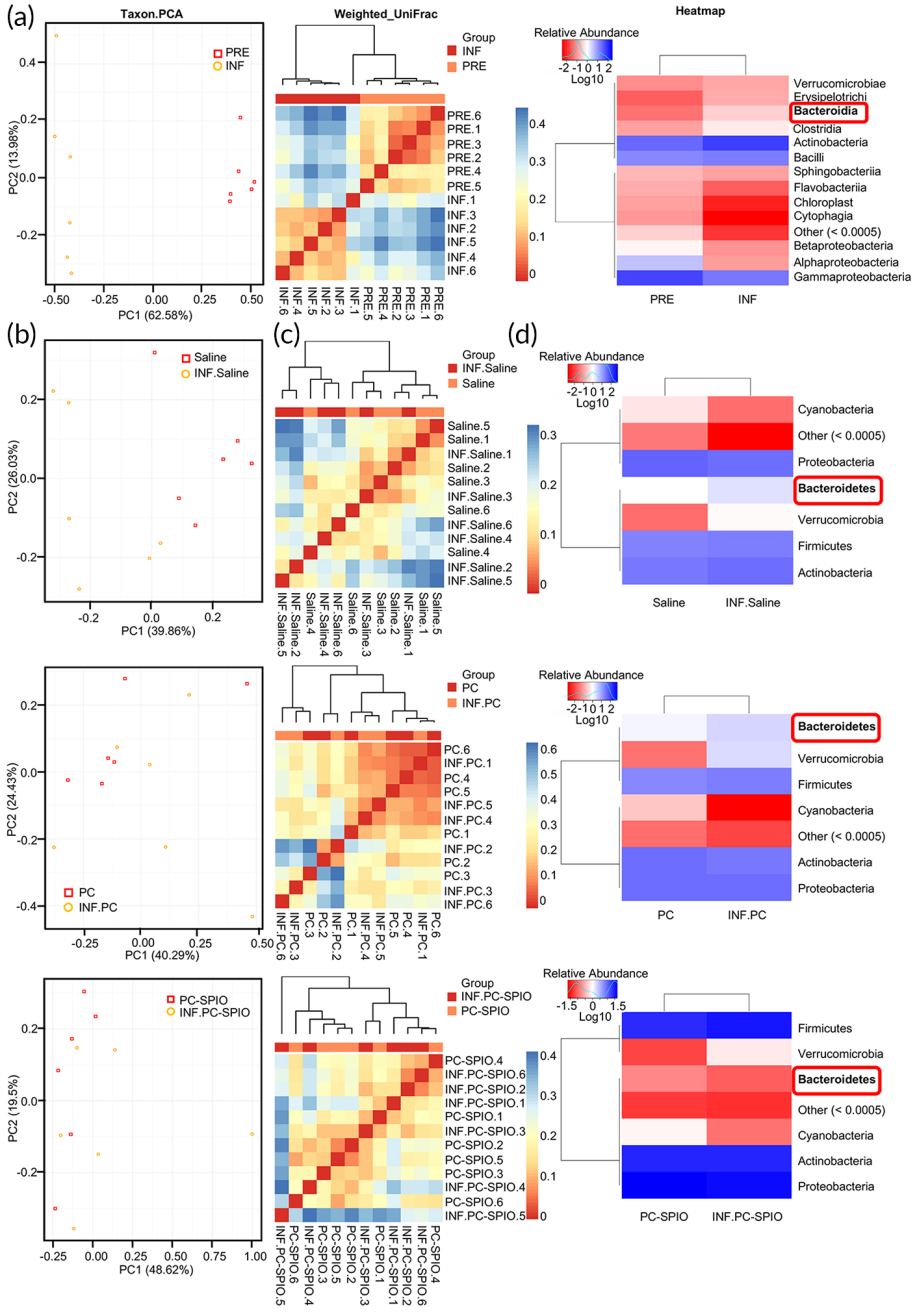
The changes in the oral microbiota induced by lipopolysaccharide (LPS) injection were not detected in the SPIO‐labeled PDLSCs (PC‐SPIO) group. (a) Comparison of the microbiota of the PRE and INF groups by 16S rRNA gene sequencing, including Taxon.PCA, Beta Weighted_Unifrac test, and Heatmap analysis (class level). (b) Taxon.PCA analysis of the microbiota from different groups of SD rats: Saline and INF.Saline; PC and INF.PC; and PC‐SPIO and INF.PC‐SPIO. (c) Beta Weighted_Unifrac test of the microbiota from different groups of SD rats: Saline and INF.Saline; PC and INF.PC; and PC‐SPIO and INF.PC‐SPIO. (d) Heatmap analysis (phylum level) of the microbiota from different groups of SD rats: Saline and INF.Saline; PC and INF.PC; and PC‐SPIO and INF.PC‐SPIO

Cumulative curves of operational taxonomic units (OTU) and OTU rank curve were provided in Figures S5 and S6. Then, the effects of LPS on the oral microbiota were detected by comparing the microbiota from normal rats before surgery (PRE group) with those that received one LPS injection and periodontal surgery (INF group). The two groups were significantly separated in the PC1 direction (Taxon) by principal components analysis (PCA). Moreover, they were obviously different according to the beta analysis (Weighted_UniFrac) (Figure 6a). Species abundance analysis showed that the content of *Bacteroidia* in the INF group was significantly higher than that in the PRE group (Figure 6a). Therefore, these results indicated that LPS markedly changed the oral microbiota by increasing *Bacteroidia*. As reported previously^31^ and confirmed in this study, LPS prevented healing and induced inflammation. It indicated that an increase of *Bacteroidia* might be related to the negative effects induced by LPS.

Subsequently, the effects of LPS on oral microbiota under PC‐SPIO injection was explored and compared with those of PC and Saline. The PCA results showed that the microbiota of the Saline group and the INF.Saline (Saline + LPS) group were obviously separated in the direction of PC1 (Taxon), while the PC‐SPIO group and INF.PC‐SPIO (PC‐SPIO + LPS) group were completely mixed. Interestingly, the condition of the PC group and the INF.PC (PC + LPS) group was intermediate between the above two, presenting as partly mixed (Figure 6b). Beta analysis (Weighted_UniFrac) showed similar trends to the PCA results (Figure 6c). Species abundance analysis showed that the content of *Bacteroidetes* in the INF.Saline group was higher than that in the Saline group, and the same was true for the INF.PC group and PC group. However, the content of *Bacteroidetes* in the INF.PC‐SPIO group was not increased compared with that in the PC‐SPIO group (Figure 6d). The results indicated that LPS‐induced negative microbiota changes when combined with the injection of Saline or PDLSCs. However, the negative changes were not detected when LPS was combined with the injection of PC‐SPIO.

The effect of periodontal defect surgery on the oral microbiota presented as an increased content of *Bacteroidia*, which was similar to the effect of LPS (Figure S7A). And a decrease of *Bacteroidia* alone was not enough to enhance periodontal bone regeneration (Figure S7B,C).

### 2.6 PC‐SPIO injection induced a decrease in *Bacteroidaceae* and an increase in *Lactobacillaceae*

**FIGURE 7:**
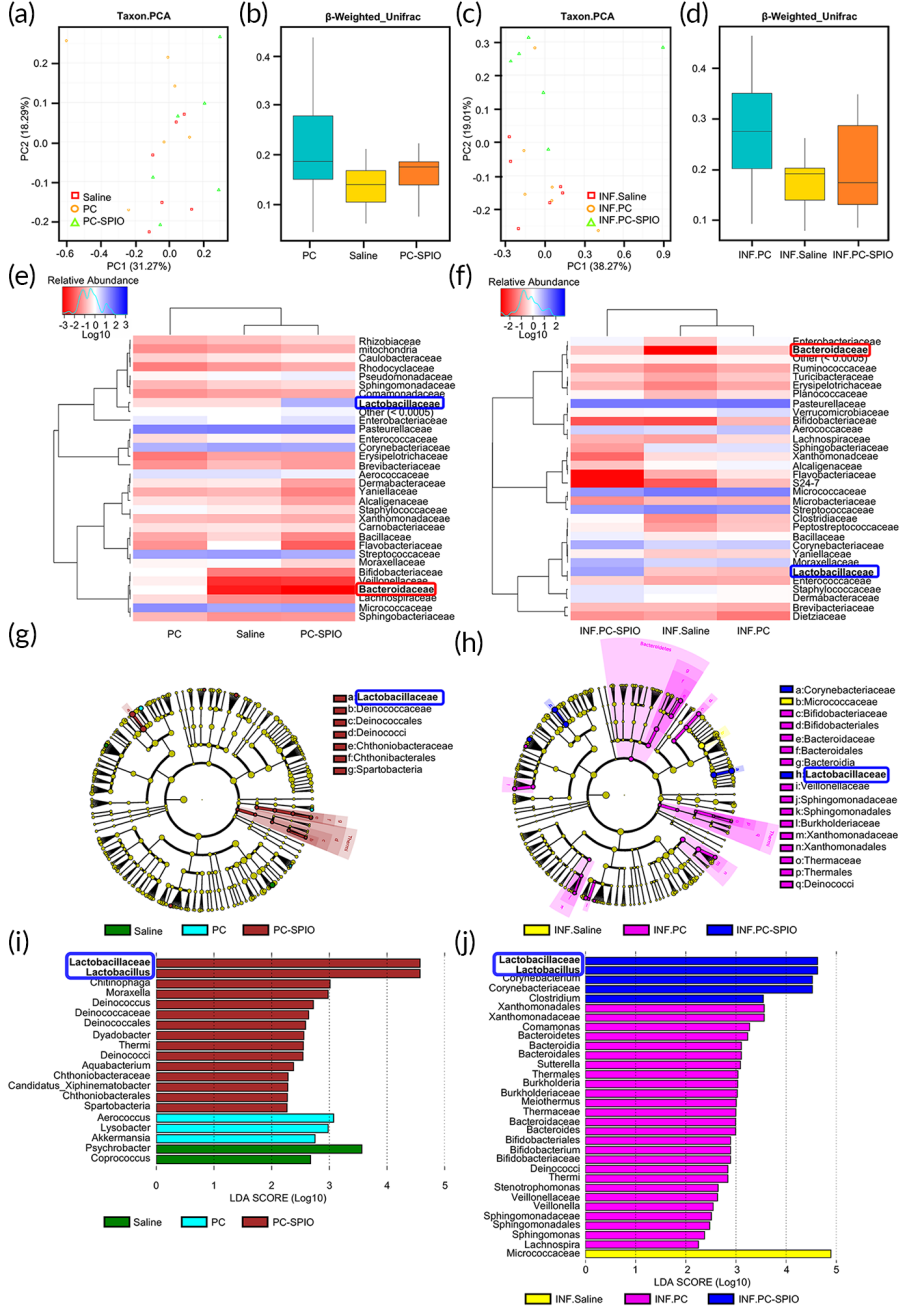
The microbiota of the superparamagnetic iron oxide (SPIO) group was different from that of the Saline and PC groups, with and without lipopolysaccharide (LPS). (a) Taxon.PCA of Saline, PC, and PC‐SPIO groups. (b) Beta Weighted_Unifrac test of the Saline, PC, and PC‐SPIO groups. (c) Taxon.PCA of the INF.Saline, INF.PC, and INF.PC‐SPIO groups. (d) Beta Weighted_Unifrac test of the INF.Saline, INF.PC, and INF.PC‐SPIO groups. (e) Heatmap analysis (family level) of the Saline, PC, and PC‐SPIO groups. (f) Heatmap analysis (family level) of the INF.Saline, INF.PC, and INF.PC‐SPIO groups. (g) LEfSe cladogram of the Saline, PC, and PC‐SPIO groups. (h) LEfSe cladogram of the INF.Saline, INF.PC, and INF.PC‐SPIO groups. (i) Linear discriminant analysis (LDA) diagram of LEfSe analysis among Saline, PC and PC‐SPIO groups. (j) LDA diagram of LEfSe analysis among INF.Saline, INF.PC and INF.PC‐SPIO groups

The microbiotas from the Saline, PC, and PC‐SPIO groups were compared. PCA (Taxon) showed that their samples were all mixed in the PC1 and PC2 directions (Figure 7a). In addition, there was no significant difference detected by beta analysis (Weighted_ UniFrac) (Figure 7b). However, when LPS was applied, INF.PC‐SPIO was separated from INF.Saline and INF.PC in the PC2 direction (Figure 7c). The difference in the beta analysis (Weighted_ UniFrac) among the INF.Saline, INF.PC, and INF.PC‐SPIO groups was significant (*p <* 0.05; Figure 7d).

Species abundance analysis showed that the content of *Bacteroidaceae* was the lowest, while that of *Lactobacillaceae* was the highest, in the PC‐SPIO group compared with those in the Saline and PC groups, with (Figure 7f) and without LPS (Figure 7e). *Bacteroidaceae* seemed not be the least abundant in the INF.PC‐SPIO group by heatmap analysis, which was caused by the computing method using the logarithm‐transformed value; the absolute content of *Bacteroidaceae* in the INF.PC‐SPIO group was 0.

Furthermore, *Lactobacillaceae* were detected as a biomarker by linear discriminant analysis effect size (LEfSe) analysis in the PC‐SPIO and INF.PC‐SPIO groups (Figure 7g,h), which was confirmed by linear discriminant analysis (LDA) (Figure 7i,j). Alpha diversity analysis (Figure S8), species richness analysis (family level) (Figure S9A,B) and key species comparison of (Top10, family level) the Saline, PC, and PC‐SPIO groups, with and without LPS (Figure S9C,D), and a Kruskal test at the family level (Figure S9E,F) was provided in Supplementary material S1. These results showed that injection of PC‐SPIO changed the oral microbiota, presenting as a decrease in *Bacteroidaceae* and an increase in *Lactobacillaceae*.

The isolation and identification of *Lactobacillus spp*. were performed (Figure S10 and Table S1). The result showed that this SPIO‐Lac had strong homology to *L. reuteri* strain (99.93%) (NCBI accession number CP041676.1). To confirm the involvement of SPIO‐Lac, which is a PC‐SPIO group biomarker in periodontal regeneration, the Spearman correlation between the abundance of *Lactobacillaceae* and the BV/TV value was calculated, using *Pasteurellaceae* as a control. The Spearman correlation coefficient between *Lactobacillaceae* and BV/TV was 0.773 (*p* < 0.01), while that of *Pasteurellaceae* was 0.059 (*p* > 0.05). The result indicated that there was a significant positive correlation between *Lactobacillaceae* and periodontal bone regeneration. Therefore, the increased abundance of SPIO‐Lac might be related to better tissue regeneration.

### 2.7 SPIO‐Lac promoted periodontal regeneration and had immunomodulatory and antibacterial properties

**FIGURE 8:**
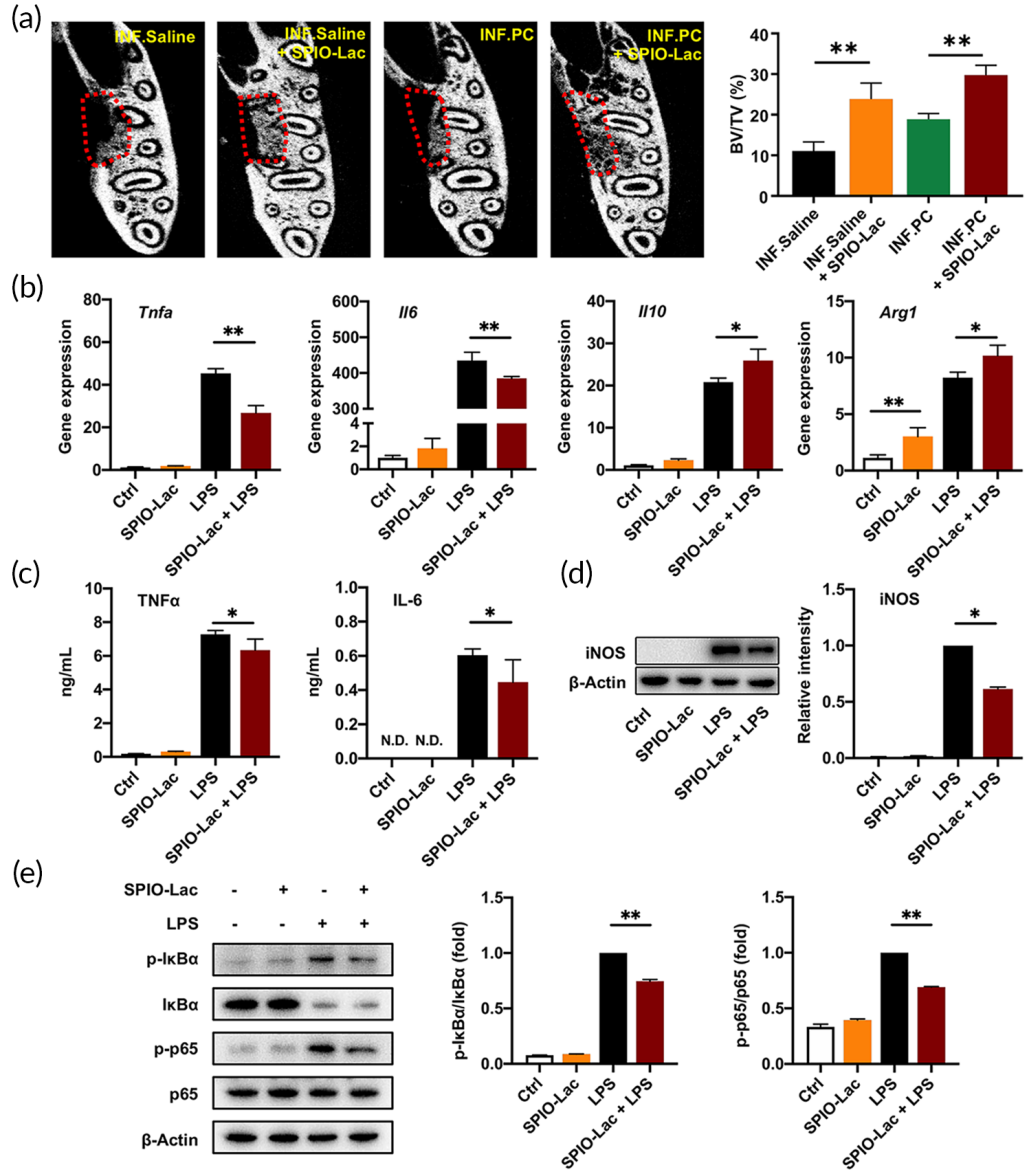
(a) Evaluation of periodontal bone regeneration promoted by SPIO‐Lac using micro‐CT and quantitative analysis of BV/TV at 2 weeks after surgery. (b) RAW264.7 cells were treated with 10^6^/ml SPIO‐Lac for 2 h and stimulated with LPS. qRT‐PCR analysis of the M1‐related gene expression levels, including *Tnfa* and *Il6*, and the M2‐related gene expression levels, including *Il10* and *Arg1* at 6 h after LPS induction. (c) Concentrations of TNFα and IL‐6 in the cell culture supernatant measured by ELISA at 24 h after LPS induction. (d) Relative protein levels of iNOS determined by western blotting at 24 h after LPS induction. (e) Western blotting and quantification analysis of total IκBα, phosphorylated (p‐) IκBα, p65, and phosphorylated (p‐) p65 at 30 min after LPS induction. (*n* = 3) (*p < 0.05, **p < 0.01)

The application of SPIO‐Lac in the INF.Saline and INF.PC groups achieved more and denser new bone at 2 weeks after surgery compared with that in the controls (*p* < 0.01; Figure 8a), indicating that SPIO‐Lac could promote periodontal bone regeneration.

In vitro, SPIO‐Lac inhibited the LPS‐induced high mRNA expression levels of *Tnfa* and *Il6* and upregulated the expression of M2‐related genes *Il10* and *Arg1* (*p <* 0.05; Figure 8b). Meanwhile, SPIO‐Lac alone did not induce higher expression levels of *Tnfa* and *Il6* (*p >* 0.05) but increased *Arg1* levels (*p <* 0.05; Figure 8b). Consistent with the mRNA expression levels, the secretion of inflammatory cytokines TNFα and IL‐6 was suppressed in the SPIO‐Lac + LPS‐treated cells compared with that in cells treated with LPS alone (*p <* 0.05; Figure 8c). Overexpression of iNOS stimulated by LPS was also ameliorated using SPIO‐Lac (*p <* 0.05; Figure 8d). When it was applied to macrophages alone, the performance of SPIO‐Lac was similar to that in the macrophage control group in these two tests (*p >* 0.05; Figure 8c,d). Moreover, the effects of SPIO‐Lac on the NF‐κB pathway were also similar to that in the control (*p* > 0.05; Figure 8e). However, SPIO‐Lac significantly inhibited the LPS‐induced NF‐κB pathway activation (*p <* 0.05; Figure 8e).

The inhibitory effect of SPIO‐Lac on the adhesion of *Porphyromonas gingivalis* (*P. gingivalis*) to human gingival fibroblasts (hGFs) was assessed (Figure S11A,B). The results indicated that SPIO‐Lac were highly adhesive to cells which prevents the adhesion of *P. gingivali*s to hGFs. And SPIO‐Lac had antimicrobial activity, not inside the bacterial cells themselves, and only partly due to its reuterin production (Figure S11C–F).

Untargeted metabolomics was performed to acquire the metabolic profiles of SPIO‐Lac supernatant against pathogens. The 21,801 peaks of positive ions and 4027 peaks of negative ions were detected and analyzed. Top 50 small molecule metabolites of SPIO‐Lac supernatant in ESI^+^ mode were amino acids, which are the basic units of polypeptides and proteins and organic acids (Figure S12).

## 3 DISCUSSION

In the present study, PDLSCs labeled by SPIO (PC‐SPIO) were applied as injections for periodontal regeneration. And we injected PC‐SPIO once a week for 4 weeks. Stem cell injection is usually injected once and achieves acceptable promoting effects for periodontal regeneration.^4^, ^6^, ^8^ Hu et al. found that a single injection of DPMSCs had lower efficiency than their sheet form.^6^ It is easy to imagine that the number of injected cells will decrease immediately following a single dose injection, as confirmed by Prussian blue staining after first injection in this study (Figure 2d). Therefore, it suggests that periodic injections are necessary to maintain the level of injected cells over the long term to improve periodontal healing.

SPIO nanoparticles were used for labeling and functional modification of PDLSCs, rather than PKH26 or luciferase.^28^, ^31^ In vivo MRI was performed at about 20 h after each injection. The results demonstrated that SPIO labeling facilitated noninvasive tracking of injected PDLSCs in the periodontal defect area. The loss and decrease of PC‐SPIO caused the image to change from hypointense (dark black) to hyperintense (bright white). The same was true for the regeneration of soft tissue, while the regeneration of hard tissue demonstrated the opposite changes. These trends were consistent with our previous report.^22^ However, the injection of PC‐SPIO was periodic in this study. The images changed back and forth between white and black because of the inflammatory reaction, PC‐SPIO injection, and bone regeneration (Figure 2b). The last MRI was performed at the 7 days after the fourth injection, thus the longevity of SPIO labeling will be detected for a longer period in future studies.

Prussian blue staining allows the evaluation of SPIO localization within the tissue microstructure. A sample without PC‐SPIO injection was prepared to prove the correlation between positive Prussian blue staining and the presence of SPIO in this study (Figure 2d). Therefore, MRI together with Prussian blue staining could be used to trace PC‐SPIO after injection. Regarding the fate of the injected cells, previous studies indicated that they mainly appeared around the injected area. However, stem cell distribution and persistence are not exclusively local when evaluating the safety of the therapy. In this study, histological staining showed the preservation of the structure and morphology of the rats' major organs, proving the biosafety of PC‐SPIO (Figure S4).

Enhanced periodontal regeneration by PDLSCs implantation has been achieved with and without a scaffold.^29^, ^30^ The underlying mechanisms are attributed to the osteo/dentinogenic differentiation potential of stem cells, the effect of inflammation inhibition (such as downregulation of TNFα and IL‐1β), the immunomodulatory capacities on macrophages and T cells, and the paracrine effect of various factors, including anti‐apoptosis, immunomodulation, and anti‐inflammation effects.^32^ PC‐SPIO achieved better regeneration results than PC alone, which probably reflected the stronger osteogenic differentiation capacity of PC‐SPIO compared with PC. It may also suggest the stronger anti‐inflammatory and repair promoting effects of PC‐SPIO compared with those of PC, which was represented by the downregulation of IL‐1β and IL‐17 and the upregulation of IL‐10 in vivo (Figure 5). SPIO is a bioactive nanoparticle with osteoinductive and immunomodulatory properties.^24^, ^25^, ^26^, ^27^ Moreover, the altered microbiota induced by PC‐SPIO might be involved in enhanced periodontal regeneration.

The abundance of *Bacteroidaceae* was decreased, while the abundance of *Lactobacillaceae* was increased in PC‐SPIO group. We hypothesized that this altered microbiota was not only a consequence of the changed environment but also a driving factor for tissue regeneration. Disturbance of the oral microbiota–ecology balance in the host usually causes a series of oral infectious diseases, including periodontitis. Meanwhile, balanced oral pathogenic bacteria and probiotics can promote wound healing by maintaining MSC homeostasis.^33^

On the one hand, a reduction in its abundance benefits periodontal regeneration. The typical pathogenic bacterium of *Bacteroidaceae* is *Bacteroides forsythus*, whose name has been changed to *Tannerella forsythia*, and belongs to the red complex of periodontal pathogens.^34^ It is frequently associated with *P. gingivalis* colonization and is detected significantly more often in sites with bleeding on probing when compared with nonbleeding sites.^35^ As a result, it is related to progressive periodontal infections, which present as loss of connective tissue and severe resorption of alveolar bone.

On the other hand, the high abundance of SPIO‐Lac, which had oral probiotic properties, such as reducing oral pathogenic bacteria counts and inhibiting their adherence to hGFs (Figure S11) should also be good for periodontal regeneration. SPIO‐Lac was identified as *L. reuteri*, which is a well‐known probiotic used to treat oral infections. *L. reuteri* plays a role in periodontal regeneration by producing a broad‐spectrum antimicrobial substance, known as reuterin,^36^ and protecting against periodontal bone loss through its anti‐inflammatory and immunomodulatory effects. Oral treatment of patients with chronic periodontitis with tablets containing probiotic strain of *L. reuteri* induced a significant reduction in TNFα, IL‐1β, and IL‐17 levels.^37^ The increase in *Lactobacillus* spp. in the SPIO group might lead to the regulation of macrophage polarization and the Th17/Treg balance to promote repair.^38^, ^39^, ^40^ Clinically, parameters including the sulcus bleeding index, the periodontal probing depth, and the clinical attachment level were improved.^41^ The adjunctive use of *L. reuteri* along with SRP achieved significantly more pocket depth reduction and attachment gain, with more *P. gingivalis* reduction.^41^ In addition, *L. reuteri* can prevent bone loss by decreasing the abundance of *Bacteriodales*.^36^

Therefore, our study indicated the existence of a distinct oral microbiota in response to a specific periodontal treatment such as PC‐SPIO injection and highlighted a potential positive role of an oral microbiota in periodontal regeneration. These results suggest the possibility of periodontal repair promotion by manipulating the oral microbiota. If scaled up to human from rat, it is expected that the injection therapy should still be effective. However, the induced changes in oral microbiota may be different, due to the differences between human and rat. More and in‐depth studies are needed.

## 4 MATERIALS AND METHODS

### 4.1 In vitro cell assays

PDLSCs were incubated with different concentrations of SPIO nanoparticles. Cell viability was evaluated using Cell Counting Kit‐8 (CCK‐8; Dojindo, Japan). The 250 μg/ml was considered as the appropriate concentration for the subsequent experiment. PDLSCs were pretreated with 250 μg/ml SPIO nanoparticles for 1 day in α‐MEM culture medium (termed PC‐SPIO), using untreated PDLSCs (termed as PC) as control.

To detect cell spreading, PC‐SPIO was observed by SEM (Phenom, Holland) at 1 day. Cell proliferation of PC‐SPIO was detected by Live/Dead cell staining (Invitrogen, USA) using inverted fluorescence microscope (Leica, Germany) at 7 days.

To test in vitro MRI labeling effects of PC by SPIO nanoparticles, PC‐SPIO were uniformly distributed in the 2% agarose solution, which later turned into a gel in a 1 ml tube. MRI presented the horizontal plane of the tube. The T2 relaxation time was measured by Image Display and Processing ParaVision 6.0.1.

To evaluate the SPIO nanoparticles in the cells, PC and PC‐SPIO were fixed by 2.5% glutaraldehyde solution at 4°C overnight for TEM (Tecnai Spirit Biotwin, USA). Furthermore, 1 × 10^5^ PC and PC‐SPIO were diluted to 5 ml after nitrolysis by 500 μl 64% nitric acid, and the concentration of iron were measured by ICP‐OES (Avio500, USA).

### 4.2 Animal experimental design and injection therapy

Periodontal defects were surgically prepared following the method of King et al.^42^ with minor modifications using specific‐pathogen‐free (SPF) male SD rats (180–200 g weight, Animal Center of Nanjing Medical University, *n* = 6). All animal procedures were reviewed and approved by the Institutional Animal Care and Use Committee of Nanjing Medical University (IACUC‐1908036; Nanjing, China). Generally, the rats were maintained in a SPF facility under controlled temperature and dark cycle, with free access to water and normal rodent food. The defect was approximately 2 mm in width, 3.5 mm in length, and 1.5 mm in depth and was about 2 mm below the alveolar crest at the buccal side of the second molars in the left mandible. After surgery, 0.1 ml saline contained 1 × 10^6^ PC or 1 × 10^6^ PC‐SPIO were injected into periodontal bone defects, using 0.1 ml saline as control.^43^ Inflammation was established by repeated injection of 10 μl of 5 mg/ml LPS from *Escherichia coli* O111:B4 (Sigma‐Aldrich, St, Louis, MO, USA),^44^ and these groups were marked as INF. Therefore, the six experimental groups were PC, PC‐SPIO, Saline, INF.PC, INF.PC‐SPIO, and INF.Saline.

*Cell injection therapy*: Cell mixture should be used as soon as possible after preparation. The 1 ml syringe with a needle of 15.5 mm in length, 0.45 mm in external diameter, and 0.25 mm in internal diameter was used. The tip of the needle was injected through the buccal mucosa into the defect up to the lingual bone wall (about 3–5 mm) at the middle of the bone defect, and drawn a little back when significant resistance was encountered. Therefore, the tip of the needle stayed at the bottom of the bone defect beneath the periosteum. Then the cell mixture was injected. It was injected slowly for 3–5 s and the tip of the needle was kept for 5–10 s after injection.^43^ The needle was withdrawn without compression. The injections were repeated once a week for four times.

*Lactobacillus* from SPIO was isolated and purified (term as SPIO‐Lac). To confirm its repair promoting effect, it was applied in INF.Saline group and INF.PC group.

### 4.3 Collection of oral microbiotas and bioinformatic analysis

The oral microbiota of periodontal defect area was collected before and after surgery. After anesthesia, the aseptic sampling swab was rubbed on the oral gingival mucosa of the periodontal defect area for 15–20 s according to the manufacturer's instructions. Then, the microbiotas on the swab were transferred to the sampling tube with universal transport medium for bacterium (Yocon Biology, China). And the samples were frozen at −80°C. 16S rRNA gene sequencing and bioinformatics analysis was done by BGI (Huada Gene Institute, China).

### 4.4 Isolation of *Lactobacillus spp*. from the microbiota

*Lactobacillus spp*. from PC‐SPIO group was isolated and purified using MRS medium (Oxoid, UK) in an anaerobic incubator (90% N_2_, 5% H_2_, and 5% CO_2_, 37°C). The round‐shape, off‐white, clear‐edged, smooth‐surfaced mono colony was picked and inoculated on a new MRS plate. The purification was repeated until the obtained colonies were single and consistent.

### 4.5 Biological effect detection of SPIO‐Lac

RAW264.7 were pretreated with 10^6^/ml heat‐inactivated SPIO‐Lac for 2 h before 30 min LPS stimulation. Then, cell protein was extracted for western blotting of IκBα, phosphorylated (p‐) IκBα, p65 and phosphorylated (p‐) p65. After 6 h LPS stimulation, RNA was extracted for qRT‐PCR analysis of the M1‐related gene expression levels, including *Tnfa* and *Il6*, and the M2‐related gene expression levels, including *Il10* and *Arg1* at 6 h after LPS induction. After 24 h LPS stimulation, concentrations of TNFα and IL‐6 in the culture supernatant were measured by ELISA (PeproTech, USA) and protein levels of iNOS were determined by western blotting.

### 4.6 Statistical analysis

All data were analyzed using the Prism 7 program (GraphPad Software Inc., La Jolla, CA, USA). Independent unpaired two‐tailed Student's *t* tests were used when comparing two groups. When more than two groups were compared, one‐way analysis of variance (ANOVA) was applied. *p* values less than 0.05 were considered statistically significant.

## 5 CONCLUSION

Although more conclusive research is needed, an altered oral microbiota induced by injections of PC‐SPIO helps periodontal bone regeneration in rats due to its probiotic properties, including anti‐inflammation and anti‐bacterial. Moreover, SPIO labeling can be used to track injected cells.

## AUTHOR CONTRIBUTIONS

**Zihan Shi:** Data curation (lead); investigation (lead); validation (equal); writing – original draft (supporting); writing – review and editing (supporting). **Lu Jia:** Data curation (lead); investigation (lead); validation (equal); writing – original draft (supporting); writing – review and editing (supporting). **Qian Zhang:** Data curation (equal); investigation (equal); validation (equal); writing – original draft (supporting); writing – review and editing (supporting). **Liuxu Sun:** Data curation (equal); investigation (equal); validation (equal); writing – original draft (supporting); writing – review and editing (supporting). **Xinyue Wang:** Investigation (supporting); writing – review and editing (supporting). **Xuan Qin:** Investigation (supporting); writing – review and editing (supporting). **Yang Xia:** Conceptualization (lead); funding acquisition (lead); project administration (lead); resources (lead); supervision (lead); validation (lead); writing – original draft (lead); writing – review and editing (lead).

## CONFLICT OF INTEREST

The authors have no conflicts of interest to declare.

### PEER REVIEW

The peer review history for this article is available at https://publons.com/publon/10.1002/btm2.10466.

## Supporting information


**Appendix S1:** Supporting Information.

## Data Availability

The data that support the findings of this study are available from the corresponding author upon reasonable request.
