# Supplementary material for: An altered oral microbiota induced by injections of superparamagnetic iron oxide nanoparticle‐labeled periodontal ligament stem cells helps periodontal bone regeneration in rats
Source: Bioeng Transl Med. 2022 Dec 13;8(3):e10466. doi: 10.1002/btm2.10466 (PMC10189485; doi:10.1002/btm2.10466)
Supplement: Supplementary file 1 — Appendix S1: Supporting Information. [file BTM2-8-e10466-s001.pdf]

Supporting information for

An altered oral microbiota induced by injections of superparamagnetic iron oxide nanoparticle-labeled periodontal ligament stem cells helps periodontal bone regeneration in rats

Zihan Shi,[1,4] Lu Jia,[1,2] Qian Zhang,[1,3] Liuxu Sun,[1] Xinyue Wang,[1] Xuan Qin,[1] Yang Xia[1,4]*

[1] Jiangsu Key Laboratory of Oral Diseases, Nanjing Medical University, Nanjing, Jiangsu 210029, People's Republic of China

[2] Department of Emergency General Dentistry, Hebei Key Laboratory of Stomatology, Hebei Medical University, Shijiazhuang, Hebei, 050017, People's Republic of China

[3] Suzhou Stomatological Hospital, Suzhou, Jiangsu, 215000, People's Republic of China

[4] Jiangsu Province Engineering Research Center of Stomatological Translational Medicine, Nanjing Medical University, Nanjing, Jiangsu 210029, People's Republic of China

[*] To whom correspondence should be addressed: Associate Prof. Yang Xia, Jiangsu Key Laboratory of Oral Diseases, Jiangsu Province Engineering Research Center of Stomatological Translational Medicine, Nanjing Medical University, Nanjing, Jiangsu 210029, China. email: xiayang@njmu.edu.cn. Fax: +86-25-86516414.

Short title: An altered oral microbiota helps periodontal bone regeneration

The PDF file includes:

Details for Materials and Methods.

Figure S1. Quantitative analysis of Prussian blue staining.

Figure S2. Effects of lipopolysaccharides (LPS) injection on periodontal tissue.

Figure S3. Histological images of H&E staining.

Figure S4. Toxic effects of SPIO-PDLSCs on main organs of rats.

Figure S5. Operational Taxonomic Units (OTU) cumulative curve.

Figure S6. OTU rank curve.

Figure S7. Effects of surgery and saline injection on rat's oral microbiota.

Figure S8. Alpha diversity of microbiota samples from Saline, PC and PC-SPIO groups with and without LPS.

Figure S9. The detection of *Lactobacillaceae* in the SPIO group with and without LPS.

Figure S10. The isolation of *Lactobacillus spp.* and identification of SPIO-Lac.

Figure S11. Effects of SPIO-Lac metabolites on *P. gingivalis* and *S. aureus.*

Figure S12. Composition detection of SPIO-Lac supernatant by untargeted metabolomics.

Table S1. Biochemical characterization results of SPIO-Lac.

Table S2. Primers sequences for quantitative reverse transcription–polymerase chain reaction (qRT-PCR).

Details for Materials and Methods

Cell culture

Isolation and identification of periodontal ligament stem cells (PDLSCs) were followed Kukreja et al.[1] The middle part of the root periodontium was scraped off with a sterile blade. Then, it was digested with 3 mg/mL type I collagenase (Sigma-Aldrich, USA) for 20 min. PDLSCs were cultured in α-minimum essential medium (α-MEM, Gibco, USA) supplemented with 10% fetal bovine serum (FBS, ScienCell, USA) and 1% penicillin/streptomycin (P/S, Gibco, USA), short as α-MEM culture medium.

Their cell surface markers were identified by flow cytometry. 2×10^6 PDLSCs of P3 generation were divided into 6 centrifuge tubes with 100 μL PBS, then 5 μL CD29, CD73, CD90, CD105, CD34 or CD45 antibodies (BioLegend, USA) were added into each tube. A tube with 100 μL PBS was used as control. All tubes were incubated on ice for 30 min away from light, and washed with PBS. Resuscitate the cells in 500 μL PBS, they were sent for flow cytometer (BD, USA) detection.[1]

Primary human gingival fibroblasts (hGFs) were isolated followed Palkowitz et al.[2] Healthy gingival tissue was chopped into small pieces and digested 20 min with 3 mg/mL type I collagenase. Primary hGFs were cultured in Dulbecco' modified Eagle's medium (DMEM, Gibco, USA) containing 10% FBS and 1% P/S at 37°C and 5% CO_2, short as DMEM culture medium.

RAW 264.7 cell line was obtained from American Type Culture Collection (ATCC, USA). RAW264.7 were cultured in DMEM culture medium.

PDLSCs and hGFs were passaged when they were 80% confluence. Passage 3-6 was used for subsequent experiments. The plan was approved by the ethics committee of Stomatological College of Nanjing Medical University (PJ2018-064-001). Consent was received from the study participants, and the guidelines outlined in the Declaration of Helsinki were followed.

Characterization of SPIO nanoparticles

SPIO nanoparticles comprising an iron oxide core and a PSC shell were obtained from Chiatai Tianqing Pharmaceutical Group Co., Ltd. (Nanjing, China). Suspension of SPIO nanoparticles was dropped on a test strip and observed by TEM (Tecnai G2 Spirit Bio TWIN, FEI, Hillsboro, OR).

CCK-8 assay

1×10^4 PDLSCs were seeded in 96-well plates and treated with SPIO nanoparticles at various concentrations for 1 and 7 days. Then, 10 μL of CCK-8 solution were added to each well and incubated at 37 °C for 2 h. The absorbance was measured at 450 nm using a microplate reader (SpectraMax M2, Molecular Devices, USA).

Cell morphology detection and live/dead staining.

To detect the effect of SPIO nanoparticles on cell morphology and proliferation, 1×10^6 PC or PC-SPIO were seeded in 48-well plates (n=3). After cultured for 7 days, PC and PC-SPIO were resined by PBS for three times, and incubated in 150 μL Calcein -AM (2 μM)/enomorphic dimer 1 (4 μM) staining solution (Invitrogen, USA) at 37 °C for 15 mins in dark. They were observed with inverted fluorescence microscope (Leica, Germany), under 490nm excitation wavelength for live cells (green fluorescence), under 530nm excitation wavelength for dead cells (red fluorescence).

In vitro cell MRI

1×10^6 PC, 1×10^5 PC-SPIO and 1×10^6 PC-SPIO were collected for MRI. The specific parameters for *in vitro* MRI detection are: Si: 1.00/1.00 mm, FOV: 8.00/3.71 cm, T2-turboRARE, 8:1. TR:3000.0 ms, TE:36.0 ms, FA:90.0 deg.

MRI, micro-CT, and histological staining of animal tissues

PC-SPIO was tracked by MRI (Biospec 7T/20 USR, German) *in vivo*. The specific parameters are: Si: 0.50/0.50 mm, FOV: 3.16/3.27 cm, T2-turboRARE, 4:1. TR: 2628.0 ms, TE:33.0 ms, FA:90.0 deg.

The animals were sacrificed 2- or 4-weeks after surgery, and the mandibles were collected for micro-CT (SkyScan 1176). DataViewer and CTAn were used to reconstruct and quantify the data. Then, the samples were decalcified in 14% EDTA, dehydrated, embedded, sectioned, and stained, including H&E, Masson, Prussian blue and IHC staining.

The width of new bone tissue was measured between its lingual boundary (red dashed line) and buccal boundary (yellow dashed line) in the periodontal defect.[3] The new bone area fraction by Masson staining[4] and positive area of Prussian blue staining[5] was analyzed by image J.

Immunohistochemical (IHC) staining

3% H_2O_2 was incubated for 5-10 min at room temperature to eliminate endogenous peroxidase activity. The sections were rinsed with distilled water and soak in PBS for 5 min. Blocked with 5-10% normal goat serum (diluted in PBS) and incubate at room temperature for 10 min. Poured off the serum and dropped primary antibody working solution and incubated 4 °C overnight. The dilution ratio of IHC staining: rabbit polyclonal to OCN (Abcam, UK, dilution ratio 1:500), rabbit polyclonal to IL-17A (Abcam, UK, dilution ratio 1:500), rabbit polyclonal to IL-1β (Immunoway, USA, dilution ratio 1:200), rabbit polyclonal to IL-10 (Proteintech, China, dilution ratio 1:200). Dropped appropriate proportion of diluted biotin-labeled secondary antibody (1%BSA-PBS dilution), and incubate at 37 °C for 10-30 min. Rinsed with PBS and added the second generation of horseradase labeled Streptomyces ovalbumin working solution, incubated at room temperature for 10 to 30 min. After color development and counterdyed, the section was then sealed with neutral gum. IHC was analyzed by Image Pro Plus.[6]

The process of 16 sRNA gene sequencing and bioinformatic analysis

Phenol extraction method was used for bacterial genomic DNA extraction. In brief, DNA was quantified by Qubit dsDNA BR Assay kit (Invitrogen, USA) using a Qubit Fluorometer, and the quality was checked by running aliquot on 1% agarose gel. 30 ng qualified genomic DNA samples and corresponding fusion primers were used to configure the polymerase chain reaction (PCR) reaction system. Agencourt AMPure XP magnetic beads were used to purify the PCR amplification products. They were dissolved in elution buffer for library building. The Agilent 2100 Bioanalyzer was used to detect the fragment range and the concentration of the library. HiSeq platform was used for sequencing. The off-machine data is filtered, and the remaining high-quality clean data is used for further analysis. The reads are spliced into tags through the overlap relationship between reads. The tags are clustered into OTU and compared with the database, species annotations. OTU rank curve and species accumulation curves were plotted with R package version3.1.1. Alpha and beta diversity were estimated by MOTHUR (v1.31.2) and QIIME (v1.8.0) at the OTU level. Sample cluster was conducted by QIIME based on UPGMA. Barplot and heatmap of different classification levels was plotted with R package v3.4.1 and R package "gplots", respectively. Principal component analysis (PCA) of OTUs was plotted with R package "age4". Partial least squares discrimination analysis (PLS-DA) was performed by R package mixOmics. Principal co-ordinates analysis (PCoA) was performed by QIIME (v1.8.0). Linear discriminant analysis effect size (LEfSe) cluster and linear discriminant analysis (LDA) analysis was conducted. Significant species or function were determined by R (v3.4.1) based on Wilcox-test or Kruskal-test.

Bacterial culture

P. gingivalis were cultured on brain heart infusion (BHI, OXOID, UK) agar (BioFroxx, Germany) plate supplemented with 5% sterile defibrinated sheep blood (DENING BIO, China), 0.5 mg/mL hemin (Aladdin, China) and 0.1 mg/mL Vitamin

K (Aladdin, China) in an anaerobic incubator (90% N_2, 5% H_2 and 5% CO_2, 37 °C). The bacterial concentration was determined by turbidimeter, and diluted to the required concentration with cell culture medium.[7]

S. aureus were cultured on Lysogeny broth (LB) agar (BioFroxx, Germany) supplemented with 5 mg/mL beef extract (Solarbio, China), 10 mg/mL peptone (Solarbio, China), 5 mg/mL sodium chloride (SCR, China) at 37 °C under aerobic conditions. The bacterial concentration was determined by turbidimeter, and diluted to 10^6 CFU/mL with LB broth.[7]

Identification of SPIO-Lac

SPIO-Lac was identified by 16S ribosomal DNA (16S rDNA) identification.[6] The amplified PCR product was analyzed using 1% agarose gel electrophoresis and sequenced by Sangon Biotech Co., Ltd. (Shanghai, China). Genomic DNA was isolated using Ezup column bacterial genomic DNA Extraction Kit (Sangon Biotech, Shanghai, China). The PCR primer pair was as follows: 27F: 5'-AGTTTGATCMTGGCTCAG-3'; 1492R: 5'- GGTTACCTTGTTACGACTT-3'. The PCR reaction was performed using 0.5 μL 20-50 ng of template DNA, and 2.5 μL buffer with Mg2+, 1 μL dNTPs, 0.2 μL Taq polymerase, 0.5 μL 10 μM of each primer and sterile ddH_2O (final volume of 25 μL). Initial denaturation was 94 °C for 4 min. Cycle denaturation, annealing and extention were 94 °C for 45 s, 55 °C for 45 s and 72 °C for 1 min respectively and for 30 cycles in total. Final extension at 72 °C for 10 min.[6] The sequence was analyzed using online Basic Local Alignment Search Tool (BLAST) in NCBI.

The morphological characterization of SPIO-Lac was observed by the Gram staining (Jiancheng Biotech, China)[8] and SEM. The Gram stain kit (Jiancheng Biotech, China) was used to characterize the morphology and properties of SPIO-Lac. The round-shape, off-white, clear-edged, smooth-surfaced mono colony was collected from MRS agar and resuspended in PBS. SPIO-Lac solution was added on glass slide and dry naturally. Dyed ammonium oxalate crystal violet for 1 min, and rinsed with

distilled water. Cover the surface with the iodized solution for 1 min, and rinsed with distilled water. Use absorbent paper to remove moisture. Added a few drops of 95% alcohol and gently shook to decolorize, rinsed with distilled water, and removed moisture after 20 s. Dyed red for 1 min, rinsed with distilled water, dried, and observed bacteria morphology under microscope.[8]

1 mL 2×10^8 CFU/mL SPIO-Lac was centrifuged at 4000 rpm/min at 4 °C for 15 min. Then SPIO-Lac suspended and fixed with 300 μL 2.5% glutaraldehyde overnight at 4 °C, rinsed by PBS, dehydrated, and dried with gradient ethanol. 10 μL solution was dripped on silicon plate, dried naturally, rinsed by PBS, dehydrated, and dried with gradient ethanol.

The biochemical characterization of SPIO-Lac was performed using the catalase activity and hydrogen peroxide hydrolysis test, nitrite reduction test, hydrogen sulfide production test, and analysis of carbohydrate fermentation profiles including lactose, maltose, galactose, and glucose. *P. gingivalis* (ATCC 33277) was used as the control.

The catalase activity and hydrogen peroxide hydrolysis test modified by Agüero et al[9] was carried out to demonstrate the presence of the enzyme catalase. The isolated and purified SPIO-Lac colony was selected from MRS agar using an inoculation loop, then placed on glass slides respectively. 1 to 2 drops of 3% hydrogen peroxide solution were added to the glass slide. The release of oxygen bubbles was considered as positive result. *P. gingivalis* was used as a negative control.

Nitrite reduction test was used to determine the ability of SPIO-Lac to reduce nitrites or free nitrogen gas.[9] Two reagents were used to reveal the presence of nitrites in the medium: reagent A (sulfanilic acid at 0.8% in 5 N acetic acid, Hopebio, China) and reagent B (α-naphthylamine 0.5% in acetic acid 5 N, Hopebio, China). The broths were inoculated with pure cultures and incubated for 48 h at 37 °C. After the incubation period 0.5 mL of reagent A was added and then 0.5 mL of reagent B. The appearance of red color in the medium indicated the presence of nitrites. *P. gingivalis* was used as a negative control.

The capacity of hydrogen sulfide production was tested.[10] The purified single colony was picked and vertically inserted into H_2S medium (Hopebio, China) with a syringe, and incubated in 37 °C for 24 h, and its color change was observed. The black lead sulfide precipitate was indicated as H_2S was produced. *P. gingivalis* was used as a negative control.

To detect analyze the carbohydrate fermentation ability of SPIO-Lac,[11] 100 μL 0.5 Mc Turbidities of the SPIO-Lac suspension was transferred to glucose, maltose, lactose, or galactose fermentation test medium (Hopebio, China). The colors of the solution were determined after 48 h of anaerobic incubation. *P. gingivalis* was used as a negative control.

Application of SPIO-Lac *in vivo*

In detail, 0.1 mL 2% Carboxymethylcellulose (CMC) contained 1×10^{10} CFU/mL SPIO-Lac was applied to the surface of periodontal bone defects, using 0.1 mL 2% CMC as control. 1 mL syringe with 1.19 mm in internal diameter irrigating needle without tip was used for SPIO-Lac application.[12]

qRT-PCR

Total RNA from cultured cells and gingival tissue was extracted using TRIzol reagent (Invitrogen, USA) and purified using PureLink RNA Mini Kit (Invitrogen, USA). Complementary DNA (cDNA) was synthesized using PrimeScript RT Master Mix (Takara, Japan). ABI QuantStudio7 (Applied Biosystems, USA) was used to analyze the relative gene expression with PowerUp SYBR Green Master Mix (Applied Biosystems, USA). Transcript expression was normalized to the Ct of the housekeeping gene GAPDH. Primer sequences for detected markers were shown in Table S2.

Western blot

Western blotting and quantification analysis were used to evaluate the effect of SPIO-Lac on protein expression. Cells were lysed in RIPA buffer with phenylmethanesulfonyl fluoride (PMSF) and phosphatase inhibitors (Beyotime, China). The proteins were separated by SDS-polyacrylamide gel electrophoresis and transferred to PVDF membranes (Millipore, USA). The membranes were blocked in 5% nonfat milk for 1 h and incubated with specific primary antibodies (1:1000) at 4 °C overnight. Antibodies to iNOS, β-actin, p65, phospho- p65, IκBα, phospho-IκBα were from Cell Signaling Technology (USA). The membranes were then washed 3 times and incubated with a 1:5000 dilution of HRP-conjugated secondary antibodies (Proteintech, China) for 1 h at room temperature. Immunolabelled proteins were visualized using ECL Prime Western blotting detection reagents (GE Healthcare, USA).

Antibacterial effects of SPIO-Lac supernatant against *Staphylococcus aureus*

Preparation of SPIO-Lac supernatant.[13] 1 mL 5×10^8 CFU/mL SPIO-Lac was incubated in 20 mL MRS broth at 37 °C for 24 h under anaerobic conditions and centrifuged at 10,000 rpm/min at 4 °C for 15 min. The supernatant was collected by 0.22 μm filter.

Preparation of SPIO-Lac reuterin. 2×10^{10} CFU/mL SPIO-Lac was washed in PBS and suspended in 2 mL glycerol for 24 h, centrifuged at 8000 rpm/min at 4 °C for 10 min.[14] The supernatant was taken and filtered by 0.22 μm bacterial filter to obtain reuterin.

Preparation of SPIO-Lac cell extracts. 5×10^8 CFU/mL SPIO-Lac was incubated in 20 mL ethyl acetate at 37 °C for 24 h under anaerobic conditions and centrifuged at 10,000 rpm/min at 4 °C for 15 min, and SPIO-Lac cell extracts was obtained.[14]

The antibacterial effects of SPIO-Lac supernatant against *Staphylococcus aureus* (*S. aureus*, ATCC 25923) were tested by growth curve detection[11] and SEM observation. For SEM observation, 1mL 2×10^8 CFU/mL *S. aureus* was treated by was by 500 μL SPIO-Lac supernatant for 18 h. Then, solution was centrifuged at 4000 rpm/min at 4 °C for 15 min. *S. aureus* was suspended and fixed with 300 μL 2.5% glutaraldehyde overnight at 4 °C, 10 μL solution was dripped on silicon plate, dried naturally, rinsed by PBS, dehydrated, and dried with gradient ethanol.

For growth curve detection, 2 mL SPIO-Lac supernatant was mixed with equal volumes of 10^6 CFU/mL *S. aureus* solution. OD of 600 nm was measured every 2 h till 18 h (n=3).[8]

Agar well diffusion method was used to compare the antibacterial effect of SPIO-Lac supernatant, SPIO-Lac reuterin, and SPIO-Lac cell extracts.[15,16] ddH_2O was used as negative control, and 10 mg/mL gentamicin was used as positive control. The LB plate was punched by Oxford cups with 6.00 ± 0.10 mm in inner diameter and 7.80 ± 0.10 mm in outside diameter. 200 μL 10^6 CFU/mL *S. aureus* solution was coated on the LB plate. After dried naturally, 200 μL solutions from each group were introduced into the wells. The diameter of inhibition zones was measured 24 h after co-culture (n=3).

To evaluate adhesion capacity, hGFs were seeded in 6-well plate at 2×10^5 cells/well. Then, hGFs were treated with 10^8 CFU SPIO-Lac or 10^8 CFU SPIO-Lac *P. gingivalis* for 1 h, respectively. Three groups were set by the addition sequence of bacteria suspension.[17,18] Protection group: 10^8 CFU SPIO-Lac pretreated hGFs for 1 h and then added 10^8 CFU *P. gingivalis* for 1 h. Replacement group: 10^8 CFU *P. gingivalis* pretreated hGFs for 1 h and then added 10^8 CFU SPIO-Lac for 1 h. Competition group: 10^8 CFU SPIO-Lac and 10^8 CFU *P. gingivalis* were added into hGFs at the same time for 1 h. After washing away the non-adherent bacteria by 1×PBS, the cells were lysed with sterile water for 1 h. For calculation of SPIO-Lac, the lysis was diluted 10^5 times by MRS medium. 300 μL of the dilution was

inoculated on MRS agar plates. The off-white colonies were counted at 1 day. For calculation of *P. gingivalis*, the lysis was diluted 10^4 times by BHI medium and 200 μL of the dilution was inoculated on BHI agar plates. The black colonies were counted at 5 days.

The Total adhesion (%) was calculated using the Equation 1:

$$Total\ adhesion\ (\%) = [\frac{Nuber\ of\ bacteria\ adhered}{Total\ number\ of\ bacteria\ added\ to\ wells}] \times 100\%$$

(Eqution1)

The Inhibition (%) were calculated using the Equation 2:

$$Inhibition\ (\%) = [\frac{A1 - A2}{A1}] \times 100\%$$

(Eqution2)

Where A1 and A2 were the percentages of *P. gingivalis* adhesion in the absence and presence of SPIO-Lac.

Composition detection of SPIO-Lac supernatant

Untargeted metabolomics was performed using liquid chromatography tandem mass spectrometry (LC-MS/MS) by Beijing Qinglian Biotech Co.,Ltd. In brief, metabolite was extracted, LC-MS analysis and metabolomic data analysis was performed.

Metabolome profiling was performed with Thermo Scientific™ Dionex™ MltiMate™ 3000 Rapid Separation LC (RSLC) system coupled to Q Exactive™ hybrid quadrupole Orbitrap mass spectrometer (Thermo Scientific^{TM}). For metabolites separation, a Waters ACQUITY UPLC BEH C8 (1.7 μm 2.1 mm×100mm) was used

in ESI+ and ESI- mode. Mobile phase A was acetonitrile/water (60/40) and mobile phase B was isopropanol/acetonitrile (90/10); both A and B contained 0.1% formic acid and 10 mmol/L ammonium formate. The gradient elution program was performed at 55 °C as follows: 0 min 98% B; 1 min 98% B; 6 min 5% B; 7 min 0% B; 14.5 min 0% B; 14.6 min 98% B; 16 min 98% B. The flow rate was 300 µL/min. MS analyses were carried out using a Q Exactive™ hybrid quadrupole Orbitrap mass spectrometer (Thermo Scientific™). Metabolites were analyzed in ESI+ and ESI- mode. The spray voltages were set as 3.5 kV, respectively, the heated capillary temperature was 300 °C. The parameters of the full mass scan were as follows: a resolution of 70000, an auto gain control target under 3×10^6, a maximum isolation time of 100 ms, and an m/z range 100-1500. The parameters of the MS/MS scan were as follows: a resolution of 17,500, an auto gain control target under 1×10^5, a maximum isolation time of 50 ms, a normalized collision energy of 10, 30 and 60 v. Then used a quality control (QC) to verify the quality of the data.

Data were collected and processed using Progenesis QI 2.3 (Waters Corporation, Milford, USA) software, and the metabolome database of Human Metabolome database (http://www.hmdb.ca/), METLIN database (https://metlin.scripps.edu) and ChemSpider database (www.chemspider.com). For different samples, data alignment was performed according to the retention time deviation of 0.2 min and the mass deviation of 5 ppm. The Coefficient of Variance (CV) value was 30%, the signal-to-noise ratio was 3, the minimum signal intensity was 100000, and the summing ions were used for peak extraction. We obtained the crude data sets comprising peak names, sample information, peak area, retention time, and quality score ratio. After deleting all the pseudo positive peaks, the data were normalized. The peaks of the same compounds were combined. Finally, the identification and quantification results of metabolites were obtained.

Figures

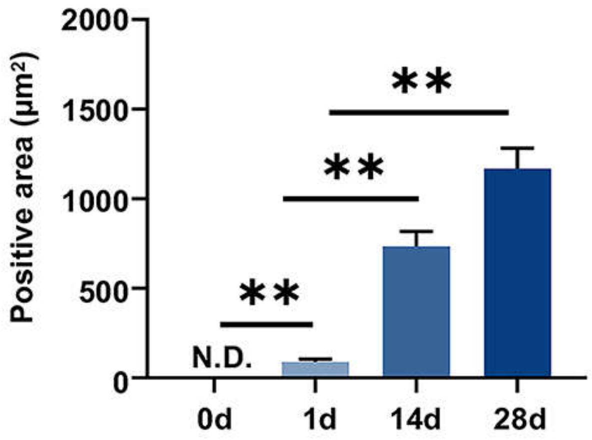

Figure S1. Quantitative analysis of Prussian blue staining. No blue stain indicating SPIO was detected without PC-SPIO injection, while the positive area of blue stains increased gradually from 1 day to 28 days with repeated PC-SPIO injections. (***p* <0.01)

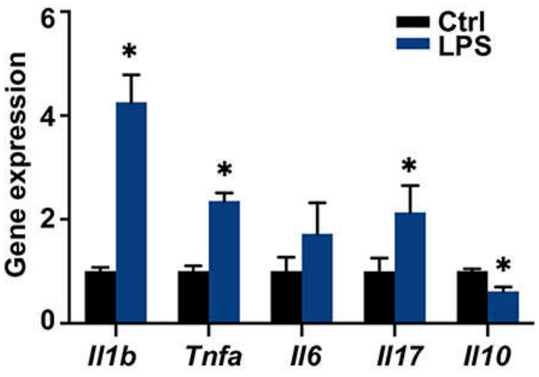

Figure S2. Effects of lipopolysaccharides (LPS) injection on periodontal tissue. RNA was extracted from gingiva 3 days after LPS injection. The expressions of *Il1β, Tnfa, Il6, Il17 and Il10* were tested by qRT-PCR. It was found that the gene expressions of *Il1β, Tnfa and Il17* were significantly increased and the gene expression of *Il10* was decreased (*p <0.05) due to LPS injection, indicating that LPS induced inflammation.

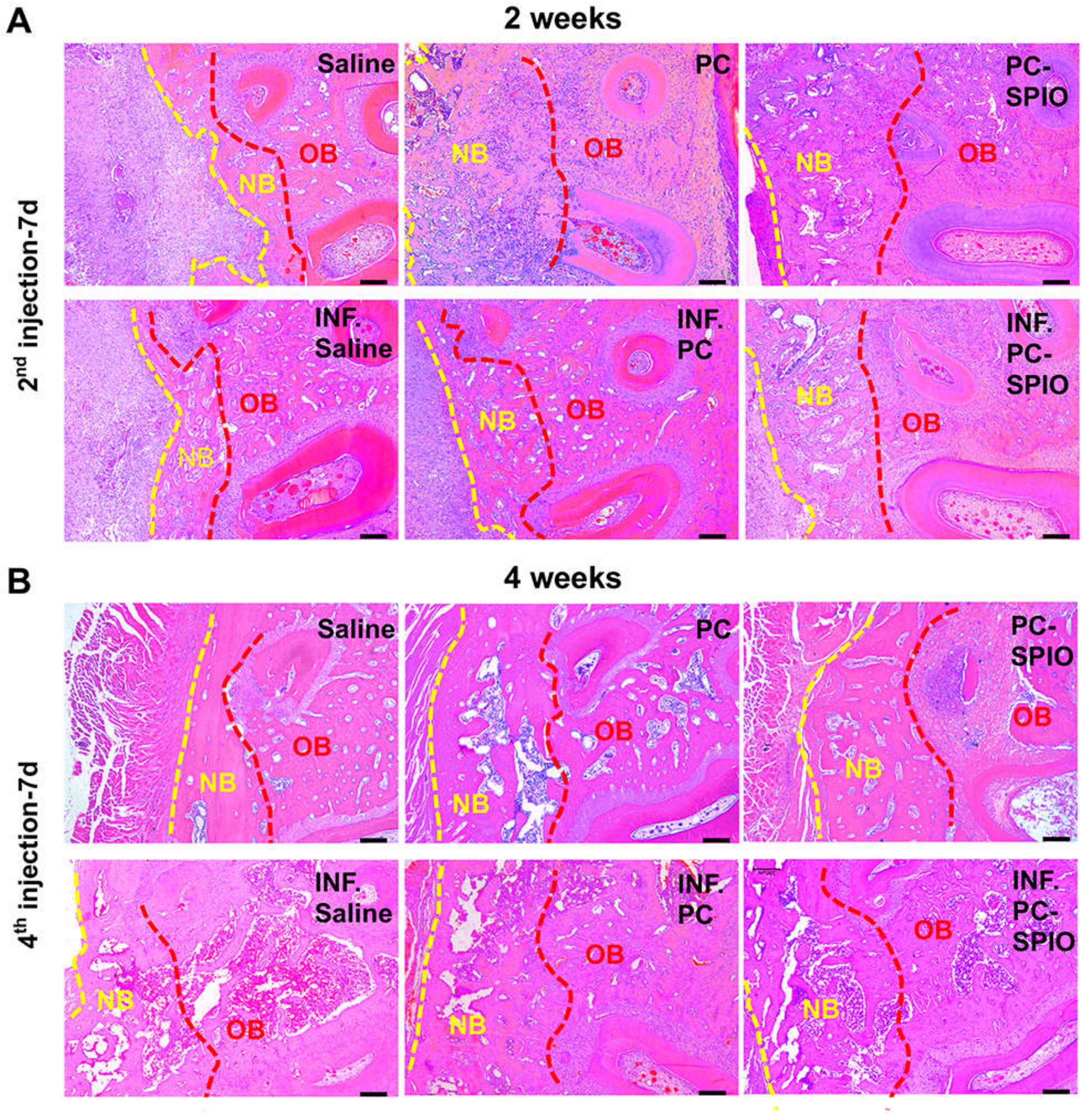

Figure S3. Histological images of H&E staining. (**A**) H&E staining of tissue samples 2 weeks after surgery. (**B**) H&E staining of tissue samples 4 weeks after surgery. The results confirmed that the regeneration in groups with LPS injection was less and poorer than those with or without LPS, the repair of the periodontal defect was in the sequence that: PC-SPIO group > PC group > Saline group, not only more but also much denser. Therefore, periodic injection of PC-SPIO had better regeneration effect on periodontal bone than PC and Saline. Scale bars, 200 μm. Abbreviations: NB, new bone; OB, original bone.

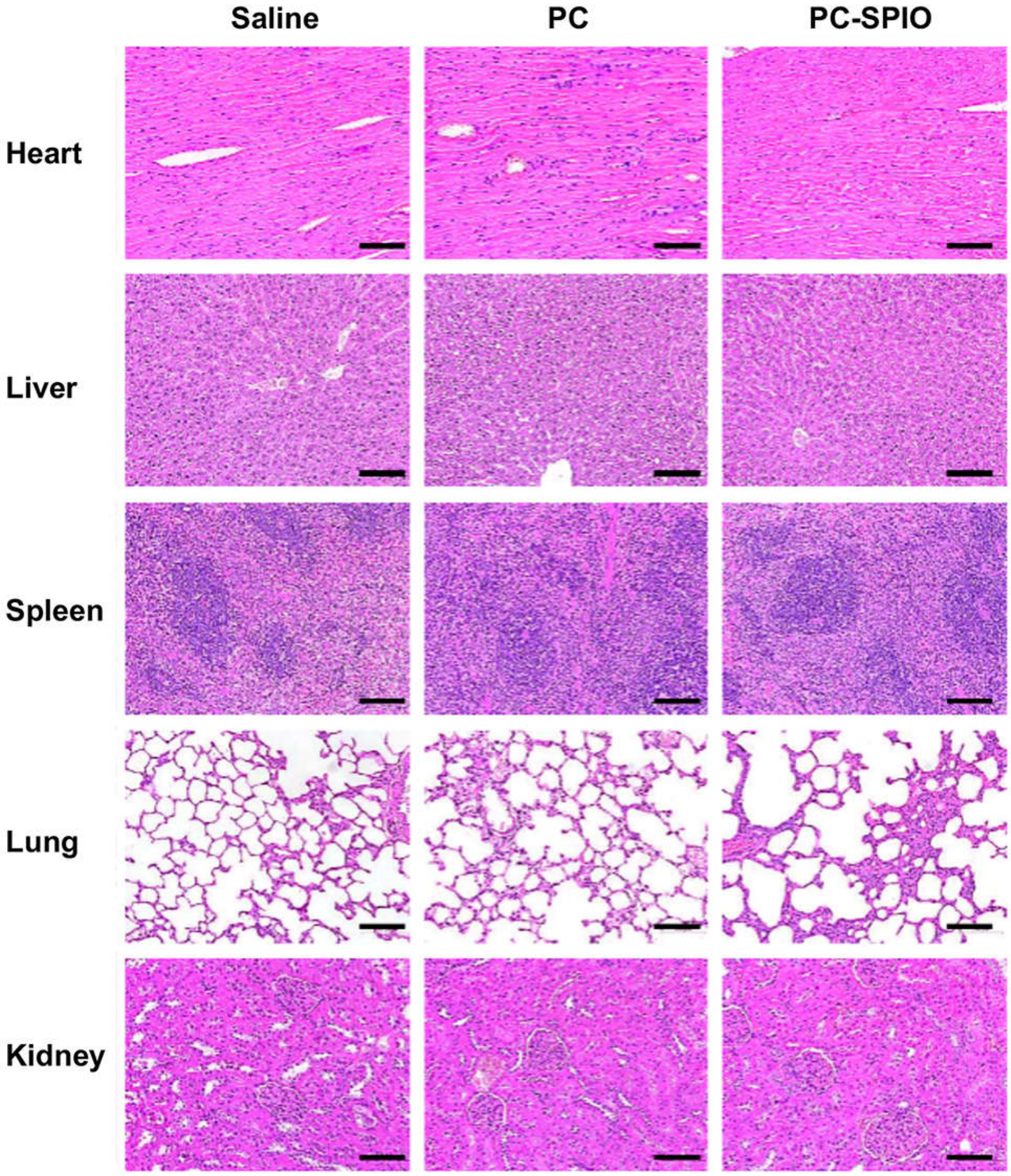

Figure S4. Toxic effects of PC-SPIO on main organs of rats. 6 weeks after the injection of PC-SPIO, the main organs including the heart, liver, spleen, lung, and kidney were sectioned and stained by H&E, compared with those from Saline and PC groups. It was found that there were no obvious differences among the samples from Saline, PC and PC-SPIO groups. These results confirmed that the periodic injection of PC and PC-SPIO had no toxic effects, and the injection therapy was biologically safe. Scale bars, 100 μm.

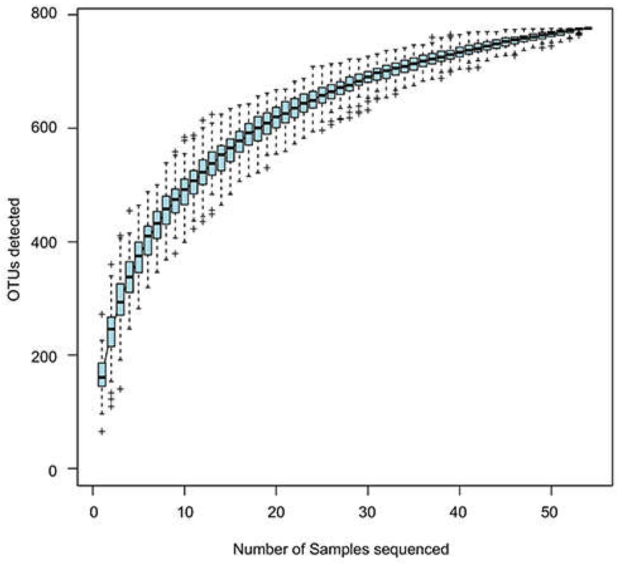

Figure S5. Operational Taxonomic Units (OTU) cumulative curve. It confirmed the adequacy of sampling.

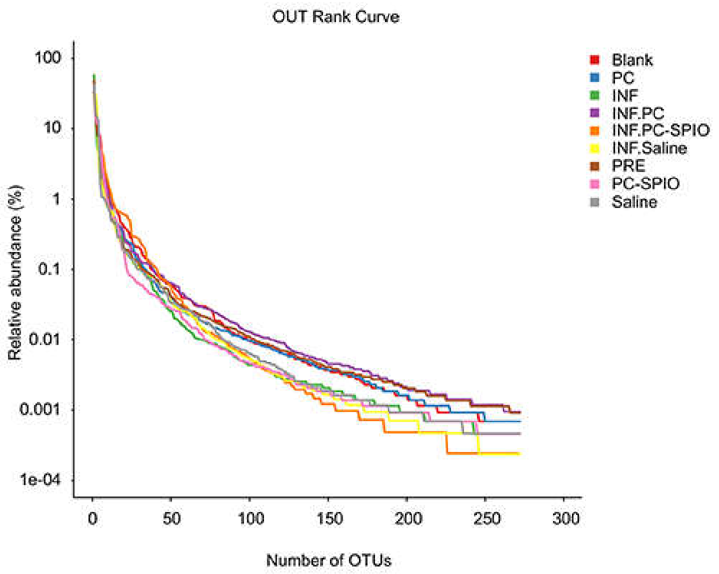

Figure S6. OTU rank curve. It showed that the species abundance of all group was similar, while some differences were noted in species evenness. The species evenness of PRE, PC, INF.PC and Blank were similar. Comparatively, the span of INF groups and PC-SPIO groups were steeper. It seemed that the species evenness was affected by LPS injection and PC-SPIO treatment.

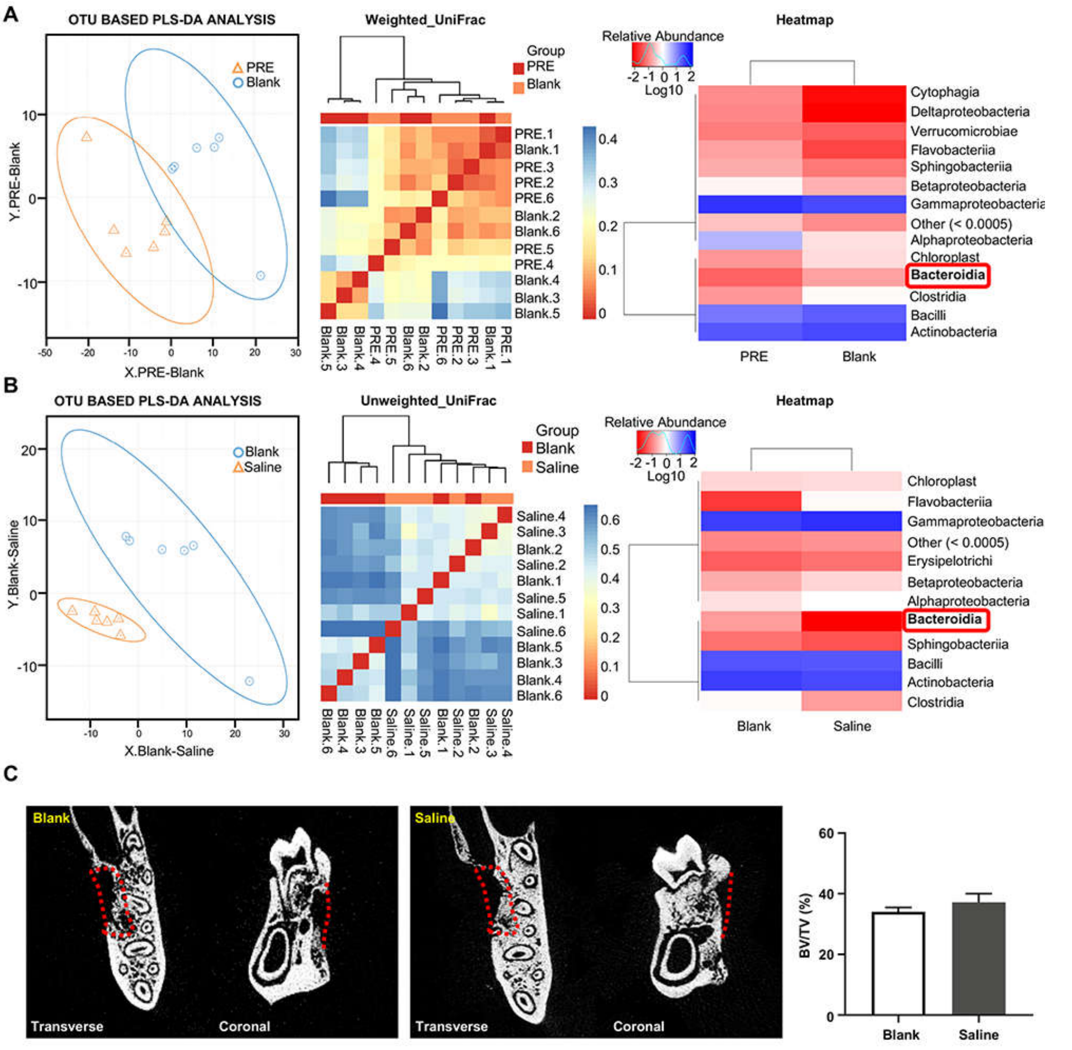

Figure S7. Effects of surgery and saline injection on rat's oral microbiota. (**A**) The comparison of microbiota from PRE group and Blank group by 16S rRNA gene sequencing, including OTU Partial Least Squares Discrimination Analysis (PLS-DA), β Weighted_Unifrac test and Heatmap analysis. PLS-DA result showed that the microbiota samples of PRE and Blank groups were well separated, and so did β analysis (Weighted_UniFrac). These results proved that the microbiota of the two groups were different. Species abundance analysis showed that the content of *Bacteroidia* in Blank group was significantly higher than that in PRE group, which was similar with LPS effect. It indicated that periodontal surgery had negative effects on microbiota, such as increase the content of *Bacteroidia*, which is a common pathogenic bacterium in oral cavity. (**B**) The comparison of microbiota from Blank group and Saline group by 16S rRNA gene sequencing, including OTU PLS-DA, β

Unweighted_Unifrac test and Heatmap analysis. PLS-DA result showed that the microbiota samples of Blank and Saline groups were well separated. It indicated that the microbiota of the two groups were different. So did the result of β analysis (Weighted_UniFrac). Species abundance analysis showed that the content of *Bacteroidia* in Saline group was obviously lower than that in Blank group. These results indicated that saline injection may be beneficial to oral microbiota, presenting as the lower content of *Bacteroidia* in Saline group than that in Blank group. (**C**) Periodontal bone regeneration 2 weeks after surgery between Blank group and Saline group was evaluated by micro-CT. And the result showed that there was no difference between the two groups ($p > 0.05$). It indicated that the decrease of *Bacteroidia* alone cannot enhance periodontal regeneration.

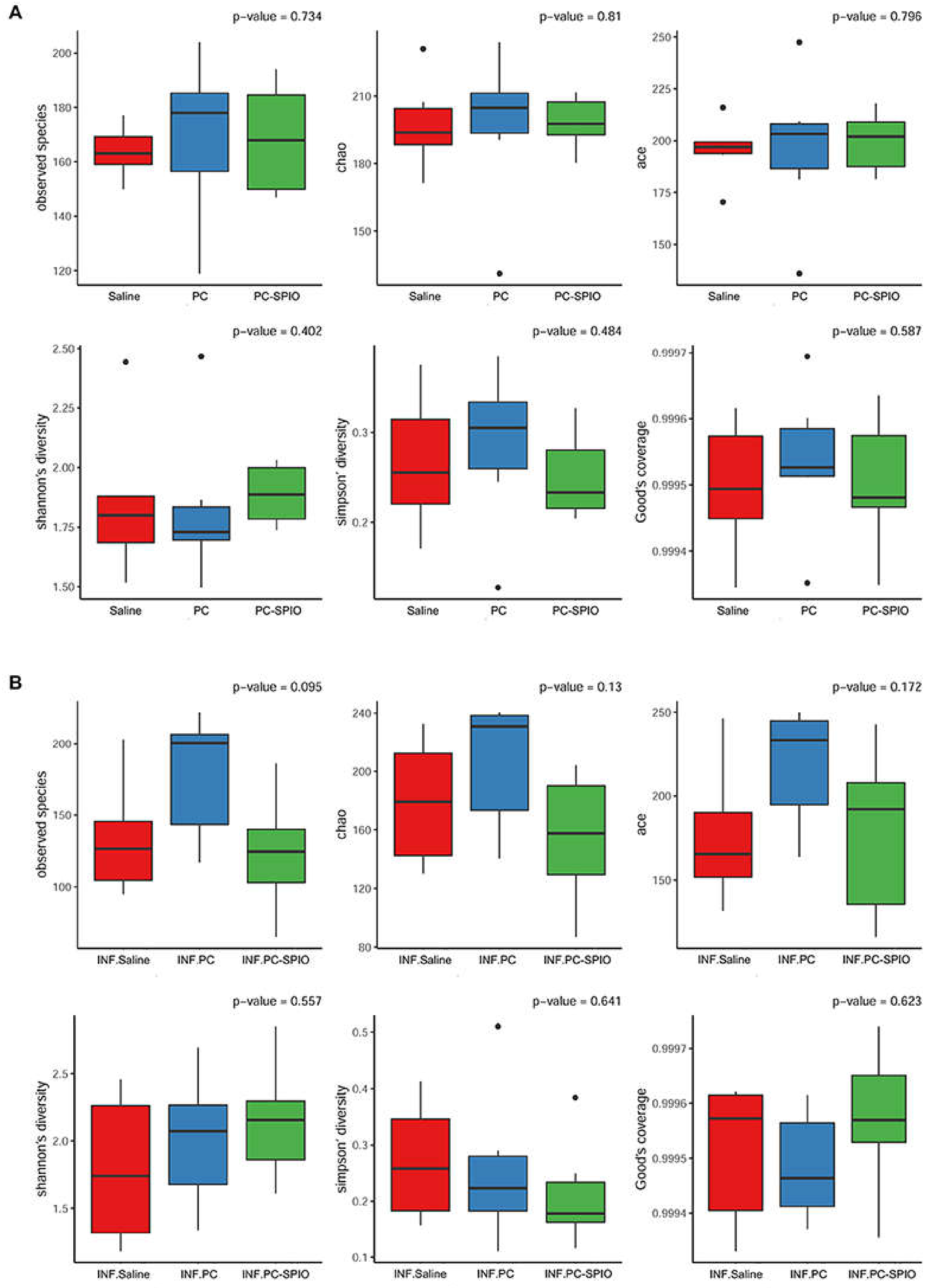

Figure S8. Alpha diversity of microbiota samples from Saline, PC and PC-SPIO groups with and without LPS. (**A**) Box diagram of alpha diversity among Saline, PC and PC-SPIO groups. (**B**) Box diagram of alpha diversity among INF.Saline, INF.PC and INF.PC-SPIO groups. These results showed that there was no significant

difference in alpha diversity among individual samples both in Saline, PC and PC-SPIO groups and INF.Saline, INF.PC and INF.PC-SPIO groups (p >0.05), which suggested that the injection therapies have no influence on species diversity within each group.

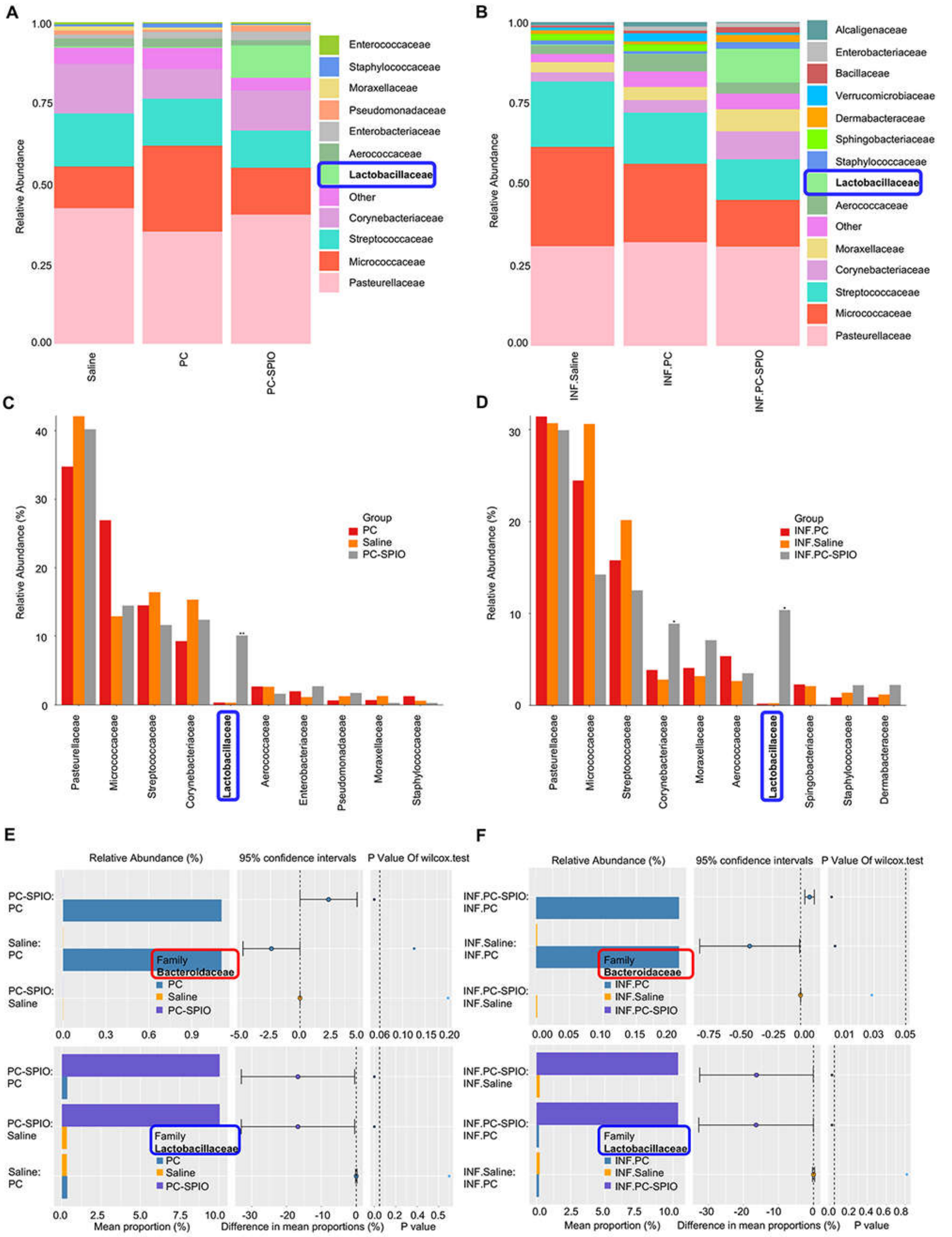

Figure S9. The detection of *Lactobacillaceae* in the SPIO group with and without LPS. (**A**) Species richness histogram (family level) among Saline, PC and PC-SPIO groups. (**B**) Species richness histogram (family level) among INF.Saline, INF.PC and INF.PC-SPIO groups. (**C**) Comparison histogram of key species differences (Top 10, family level) among Saline, PC and PC-SPIO groups. (**D**) Comparison histogram of key species differences (Top 10, family level) among INF.Saline, INF.PC and INF.PC-SPIO groups. (**E**) Comparison of the microbiotas from the Saline, PC, and

PC-SPIO groups using the Kruskal test (family level). (**F**) Comparison of microbiotas from the INF.Saline, INF.PC, and INF.PC-SPIO groups using the Kruskal test (family level). These results showed that the abundance of *Lactobacillaceae* was higher in the PC-SPIO and INF.PC-SPIO groups than in the other groups ($p < 0.05$), and proved that the PC-SPIO group had the lowest *Bacteroidaceae* content and the highest *Lactobacillaceae* content compared with the other groups, with and without LPS. Therefore, it suggested that injection of PC-SPIO changed the oral microbiota, presenting as a decrease in *Bacteroidaceae* and an increase in *Lactobacillaceae.*

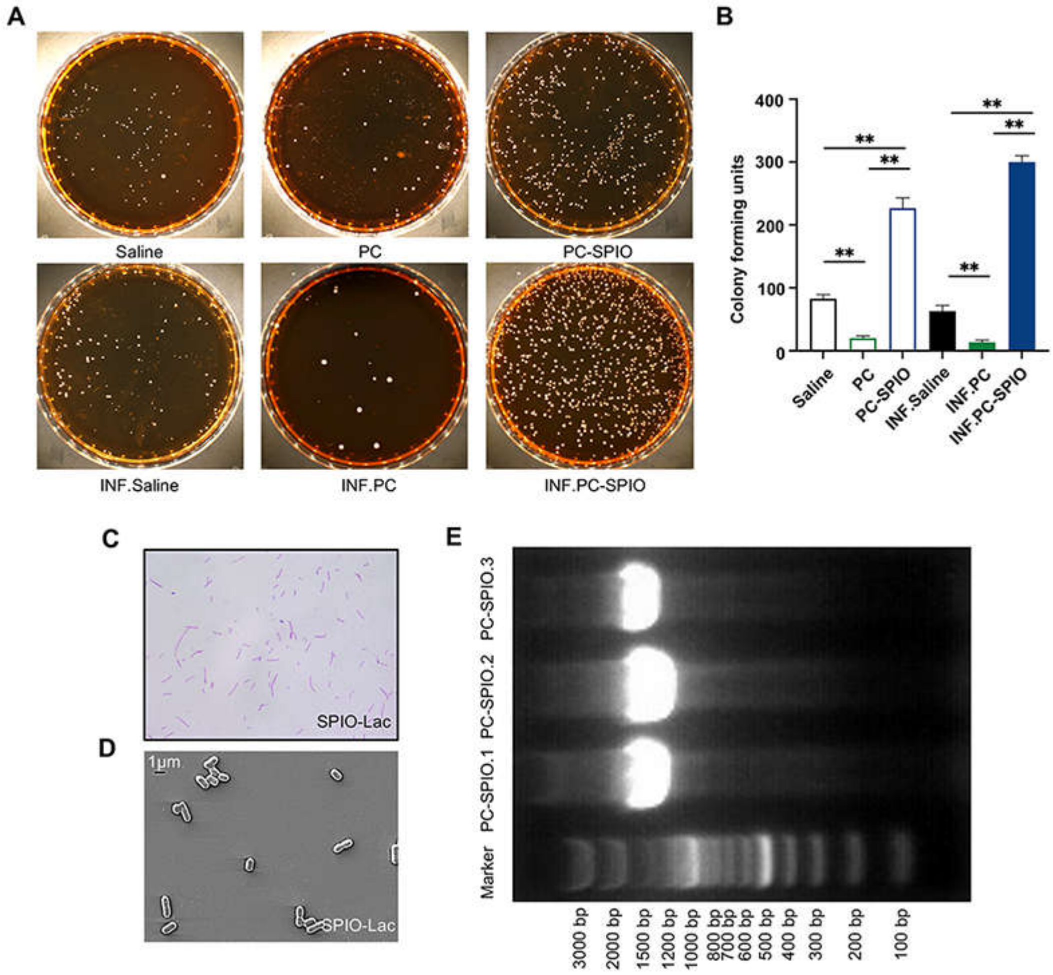

Figure S10. The isolation of *Lactobacillus spp.* and identification of SPIO-Lac. (**A**) Isolation of *Lactobacillus spp.* from microbiotas of the Saline, PC, and PC-SPIO groups, with and without LPS, using De Man, Rogosa and Sharpe (MRS) medium. (**B**) Quantitative calculation of colony formation units. More round, milky white, clear edge, and smooth surface bacterial colonies were observed in samples from the PC-SPIO group (SPIO-Lac) than in those from the Saline and PC groups, with and without LPS. (**C**) Gram staining of SPIO-Lac. SPIO-Lac is a kind of blue-violet, Gram-positive rod-shaped bacteria in chain arrangement. (**D**) SEM images of SPIO-Lac. SPIO-Lac is a rod-shaped bacterium, which coincides with the morphology of *Lactobacillus reuteri* (*L. reuteri*).[19,20] (**E**) Electrophoresis results of SPIO-Lac. (n = 3) (**$p < 0.01$).

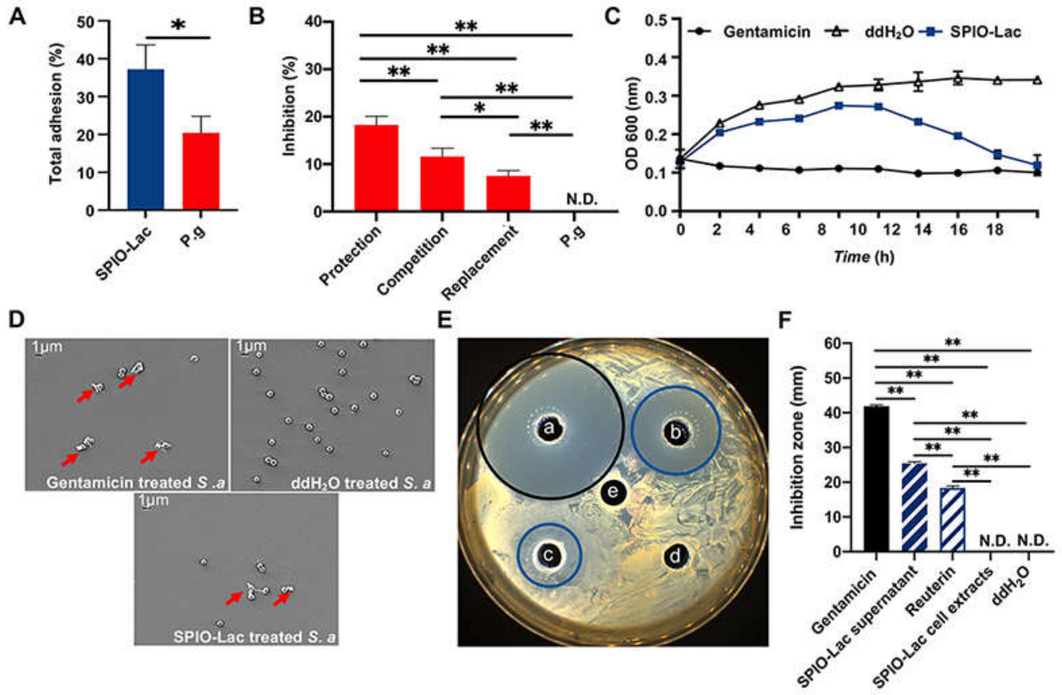

Figure S11. Effects of SPIO-Lac metabolites on *P. gingivalis* and *S. aureus*. (**A**) Adhesion (%) of SPIO-Lac and *P. gingivalis* to hGFs. (**B**) Inhibition (%) of *P. gingivalis* to hGFs by SPIO-Lac. SPIO-Lac presented stronger adhesion ability than *P. gingivalis*, and Protection group showed higher inhibition rate than Replacement and Competition groups. And inhibition rate of Competition group was higher than Replacement group. (**C**) Effect of the SPIO-Lac supernatant on the growth of *S. aureus*. It showed that the growth of *S. aureus* was inhibited by SPIO-Lac supernatant. (**D**) SEM images of SPIO-Lac treated *S. aureus*. Damaged bacteria cells (red arrows) presenting as cell shrinkage and rupture and perforation of cell membranes were observed in SPIO-Lac supernatant treated *S. aureus*. (**E**) Inhibition zone assay against *S. aureus* induced by the SPIO-Lac supernatant, reuterin production of SPIO-Lac and SPIO-Lac cell extracts. Inhibition zones were observed in the supernatant and reuterin groups. (**F**) Quantitative analysis of the inhibition zone. The zone size was in the order: gentamicin > supernatant > reuterin > cell extracts = ddH_2O. These results indicated that SPIO-Lac had antimicrobial activity, which is not inside the bacterial cells themselves, and only partly due to its reuterin production. In (**C**), (**D**) (**E**) and (**F**), the negative control was ddH_2O, and the positive control was 10 mg/mL gentamicin. (n = 3) (*$p < 0.05$, **$p < 0.01$)

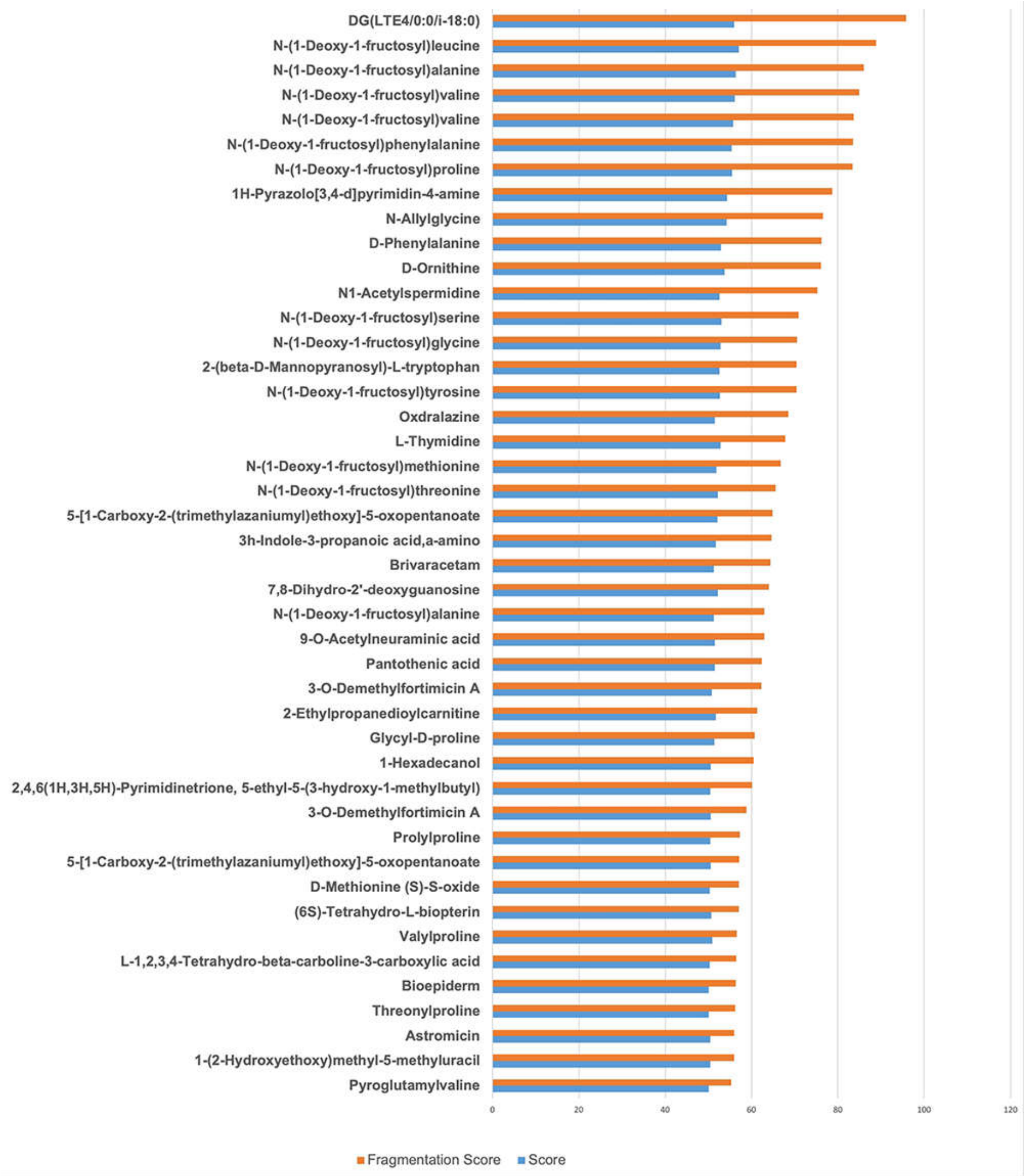

Figure S12. Composition detection of SPIO-Lac supernatant by untargeted metabolomics. Fragmentation score and score are used to ranking top 50 substances of SPIO-Lac supernatant, the result suggested possible antimicrobial components in SPIO-Lac supernatant.

References

1. Kukreja BJ, Bhat KG, Kukreja P, et al. Isolation and Immunohistochemical Characterization of Periodontal Ligament Stem Cells: A Preliminary Study. *J Indian Soc Periodontol*. 2021;25:295–299.

2. Palkowitz AL, Tuna T, Bishti S, et al. Biofunctionalization of Dental Abutment Surfaces by Crosslinked ECM Proteins Strongly Enhances Adhesion and Proliferation of Gingival Fibroblasts. *Adv. Healthcare Mater*. 2021;10:2100132.

3. Deng N, Sun J, Li Y, et al. Experimental Study of RhBMP-2 Chitosan Nano-Sustained Release Carrier-Loaded PLGA/NHA Scaffolds to Construct Mandibular Tissue-Engineered Bone. *Archives of Oral Biology*. 2019;102:16–25.

4. Coda AR, Anzilotti S, Boscia F, et al. In Vivo Imaging of CNS Microglial Activation/Macrophage Infiltration with Combined [18F]DPA-714-PET and SPIO-MRI in a Mouse Model of Relapsing Remitting Experimental Autoimmune Encephalomyelitis. *Eur J Nucl Med Mol Imaging*. 2021;48:40–52.

5. Xu F, Ren ZX, Zhong XM, et al. Intrauterine Inflammation Damages Placental Angiogenesis via Wnt5a-Flt1 Activation. *Inflammation*. 2019;42:818–825.

6. Kaur R, Tiwari SK, Identification and Characterization of a Halocin - producing Haloarchaeon Isolated from Pachpadra Salt Lake. *Lett. Appl. Microbiol*. 2020,71:620–626.

7. Boaro LCC, Campos LM, Varca GHC, et al. Antibacterial Resin-Based Composite Containing Chlorhexidine for Dental Applications. *Dental Materials*. 2019;35:909–918.

8. Bazireh H, Shariati P, Azimzadeh JS, et al. Isolation of Novel Probiotic Lactobacillus and Enterococcus Strains From Human Salivary and Fecal Sources. *Front Microbiol*. 2020;11:597946

9. Agüero NL, Frizzo LS, Ouwehand AC, et al. Technological Characterisation of Probiotic Lactic Acid Bacteria as Starter Cultures for Dry Fermented Sausages. *Foods*. 2020;9:596.

10. Robert BS, Murray EGD, Nathan RS. *Bergey's manual of determinate bacteriology* ,7th ed. Baltimore: The Williams and Wilkins Company; 1957.

11. Sun YD, Zhu YX, Zhang X, et al. Role of Cholesterol Conjugation in the Antibacterial Photodynamic Therapy of Branched Polyethylenimine-Containing Nanoagents. *Langmuir*. 2019;35:14324–14331.

12. Blasco-Baque V, Garidou L, Pomié C, et al. Periodontitis Induced by Porphyromonas Gingivalis Drives Periodontal Microbiota Dysbiosis and Insulin Resistance via an Impaired Adaptive Immune Response. *Gut*. 2017;66:872–885.

13. Geraldo BMC, Batalha MN, Milhan NVM, et al. Heat-killed Lactobacillus Reuteri and Cell-free Culture Supernatant Have Similar Effects to Viable Probiotics during Interaction with Porphyromonas Gingivalis. *J Periodont Res*. 2020;55:215–220.

14. Yang KM, Kim JS, Kim HS, et al. Lactobacillus Reuteri AN417 Cell-Free Culture Supernatant as a Novel Antibacterial Agent Targeting Oral Pathogenic Bacteria. *Sci Rep*. 2021;11:1631.

15. Hamida RS, Ali MA, Goda DA, et al. Lethal Mechanisms of Nostoc-Synthesized Silver Nanoparticles Against Different Pathogenic Bacteria. *IJN*. 2020;15:10499–10517.

16. Kadian S, Manik G, Das N, et al Synthesis, Characterization and Investigation of Synergistic Antibacterial Activity and Cell Viability of Silver–Sulfur Doped Graphene Quantum Dot (Ag@S-GQDs) Nanocomposites. *J. Mater. Chem. B*. 202;8:3028–3037.

17. Hashemi SMB, Jafarpour D, Jouki M. Improving Bioactive Properties of Peach Juice Using Lactobacillus Strains Fermentation: Antagonistic and Anti-Adhesion Effects, Anti-Inflammatory and Antioxidant Properties, and Maillard Reaction Inhibition. *Food Chemistry*. 2021;365:130501.

18. Hojjati M, Behabahani BA, Falah F. Aggregation, Adherence, Anti-Adhesion and Antagonistic Activity Properties Relating to Surface Charge of Probiotic Lactobacillus Brevis Gp104 against Staphylococcus Aureus. *Microb Pathog*. 2020;147:104420.

19. Rajab S, Tabandeh F, Shahraky MK, et al. The Effect of Lactobacillus Cell Size on Its Probiotic Characteristics. *Anaerobe*. 2020;62:102103.

20. Wasfi R, Abd EOA, Zafer MM, et al. Probiotic Lactobacillus Sp. Inhibit Growth, Biofilm Formation and Gene Expression of Caries-Inducing Streptococcus Mutans. *J. Cell. Mol. Med*. 2018;22:1972–1983.

Tables

Table S1. Biochemical characterization results of SPIO-Lac.

	SPIO-Lac	*P. gingivalis*
Catalase activity and hydrogen peroxide hydrolysis	(-)	(-)
Nitrite reduction test	(-)	(-)
H_2S production test	(-)	(-)
Glucose fermentation test	(+)	(-)
Maltose fermentation test	(+)	(-)
Lactose fermentation test	(+)	(-)
Galactose fermentation test	(+)	(-)

Table S2. Primers sequences for quantitative reverse transcription–polymerase chain reaction (qRT-PCR).

Gene	Forward Primer sequences (5′-3′)	Reverse Primer sequences (5′-3′)
Tnfa (M)	ATGTCTCAGCCTCTTCTCATTC	GCTTGTCACTCGAATTTTGAGA
Il6 (M)	CTTCTTGGGACTGATGCTGGTGAC	AGGTCTGTTGGGAGTGGTATCCTC
Il10 (M)	TTCTTTCAAACAAAGGACCAGC	GCAACCCAAGTAACCCTTAAAG
Arg1 (M)	TTGGGTGGATGCTCACACTG	GTACACGATGTCTTTGGCAGA
Gapdh (M)	CAACTCCCTCAAGATTGTCAGCAA	GGCATGGACTGTGGTCATGA
Il1b (R)	CTCACAGCAGCATCTCGACAAGAG	TCCACGGGCAAGACATAGGTAGC
Il6 (R)	GAGACTTCCAGCCAGTTGCC	ACTGGTCTGTTGTGGGTGGTA
Tnfa (R)	ATGGGCTCCCTCTCATCAGTTCC	GCTCCTCCGCTTGGTGGTTTG
Il10 (R)	CTGCTCTTACTGGCTGGAGTGAAG	TGGGTCTGGCTGACTGGGAAG
Il17 (R)	CTGTTGCTGCTACTGAACCTGGAG	CCTCGGCGTTTGGACACACTG
Gapdh (R)	TCTCTGCTCCTCCCTGTTC	ACACCGACCTTCACCATCT

M = Mus musculus, R = Rattus norvegicus
